# Milk intake across adulthood and muscle strength decline from mid- to late life: the MRC National Survey of Health and Development

**DOI:** 10.1017/S0007114522001799

**Published:** 2023-03-14

**Authors:** Antoneta Granic, Rachel Cooper, Richard M. Dodds, Susan J. Hillman, Avan A. Sayer, Sian M. Robinson

**Affiliations:** 1 AGE Research Group, Translational and Clinical Research Institute, Faculty of Medical Sciences, Newcastle University, Newcastle upon Tyne, UK; 2 NIHR Newcastle Biomedical Research Centre, Newcastle upon Tyne Hospitals NHS Foundation Trust and Newcastle University, Newcastle upon Tyne, UK; 3 Department of Sport and Exercise Sciences, Musculoskeletal Science and Sports Medicine Research Centre, Manchester Metropolitan University, Manchester, UK

**Keywords:** Skeletal muscle, Grip strength, Probable sarcopenia, Milk, Reduced-fat milk, full-fat milk, Cohort study, Ageing

## Abstract

Milk is a source of several nutrients which may be beneficial for skeletal muscle. Evidence that links lower milk intake with declines in muscle strength from midlife to old age is lacking. We used data from the Medical Research Council National Survey of Health and Development to test sex-specific associations between milk consumption from age 36 to 60–64 years, low grip strength (GS) or probable sarcopenia, and GS decline from age 53 to 69 years. We included 1340 men and 1383 women with at least one measure of both milk intake and GS. Milk intake was recorded in 5-d food diaries (aged 36, 43, 53 and 60–64 years), and grand mean of total, reduced-fat and full-fat milk each categorised in thirds (T1 (lowest) to T3 (highest), g/d). GS was assessed at ages 53, 60–64, and 69 years, and probable sarcopenia classified at the age of 69 years. We employed logistic regression to examine the odds of probable sarcopenia and multilevel models to investigate decline in GS in relation to milk intake thirds. Compared with T1, only T2 (58·76–145·25 g/d) of reduced-fat milk was associated with lower odds of sex-specific low GS at the age of 69 years (OR (95 % CI): 0·59 (0·37, 0·94), *P* = 0·03). In multilevel models, only T3 of total milk (≥ 237·52 g/d) was associated with stronger GS in midlife in men (*β* (95 % CI) = 1·82 (0·18, 3·45) kg, *P* = 0·03) compared with T1 (≤ 152·0 g/d), but not with GS decline over time. A higher milk intake across adulthood may promote muscle strength in midlife in men. Its role in muscle health in late life needs further examination.

Recent evidence from nutritional epidemiology and intervention studies supports a protective role of nutrient-rich whole foods in skeletal muscle ageing^(1)^. Milk is a whole food that, as part of a healthy diet throughout the life course, provides a combination of nutrients and non-nutrients beneficial for health^(2)^. Specifically, milk is a source of fast and slow digestible proteins and contains 20 % whey and 80 % of caseins, that have been shown to stimulate muscle protein synthesis via the mammalian target of rapamycin pathway, alone or in combination with resistance exercise, in both young and older adults^(3,4)^. Whilst whey proteins are rapidly digested and release essential amino acids such as leucine – the main regulator of muscle protein synthesis^(5)^ – casein proteins are absorbed slowly and may support extended hyperaminoacidemia prolonging the anabolic response^(6)^. We have recently hypothesised about the myoprotective potential of other milk constituents beyond pro-anabolic effects of milk proteins, including antioxidative, anti-inflammatory and immunomodulating capacity of milk bioactive components^(7)^, which may act cumulatively and synergistically in increasing the importance of milk for skeletal muscle health in older adults. For example, several milk-derived peptides, minerals, lipids and fatty acids have antioxidative and anti-inflammatory properties, thus contributing to the antioxidant capacity of a healthy diet by reducing oxidative damage to key organelles and molecules in ageing myofibres^(8)^ and attenuating inflammaging – a chronic low-grade inflammation that underlies the ageing process and contributes to the pathology of many age-related diseases, including those of muscle^(9–13)^.

Cellular processes of oxidative stress and inflammation have been implicated in the pathophysiology of sarcopenia^(11)^, a condition characterised by a progressive and generalised loss of skeletal muscle strength and mass^(12)^. Based on the current European consensus definition of sarcopenia, reduced muscle strength – assessed by grip strength (GS)^(12)^ or chair stands^(13)^ – is the primary indicator of probable sarcopenia (e.g. defined as sex-specific low GS) in older adults, which can occur both acutely (e.g. after hospitalisation with an acute illness) or progressively (e.g. with ageing). GS, a primary and easily obtained measure of muscle strength, increases steadily across early adulthood, peaks in the third or fourth decade of life, plateaus thereafter, and declines rapidly after the fifth decade in both men and women^(14,15)^. Midlife is recognised as a critical period of life when individuals experience a change in muscle strength from relative stability to progressive decline^(15)^, whilst better midlife health^(16)^ and healthier lifestyle (e.g. healthier diets) have been linked to better muscle health and function in later life^(17)^. Investigating the variability in muscle strength (GS) and associated modifiable lifestyle factors such as milk intake during this period of life could inform primary prevention strategies for sarcopenia.

We have recently highlighted a gap in knowledge in nutritional epidemiology from observational studies, that is the role of whole foods, including milk, in muscle health (defined in terms of muscle strength, mass, function and sarcopenia), and utilising a life course approach^(1,7)^. Although nutrition plays a key role in several disorders of muscle, surprisingly little is known about how nutrient-rich foods (e.g. milk, meat, seafood, eggs, whole grains and legumes) that are consumed over adulthood and provide a mix of beneficial micro- and macronutrients and biologically active components^(18)^ may influence muscle health parameters in late life. In particular, there is a lack of prospective studies investigating the association between milk intake across adulthood and sex-specific changes in muscle strength from mid- to late life – here midlife being identified as a starting point of GS decline^(15)^ in both sexes. Our objective was to address this important evidence gap, by using available longitudinal data from the Medical Research Council (MRC) National Survey of Health and Development (NSHD) to test whether milk intake from the age of 36 to 60–64 years was associated with risk of probable sarcopenia (i.e. sex-specific low grip GS) and GS decline from the age of 53 to 69 years in men and women.

## Materials and methods

### Study population

The MRC NSHD, also known as the 1946 British Birth Cohort, is a social-class-stratified random sample of all singleton births in England, Wales and Scotland during the first week of March 1946. The 5362 participants (2815 male and 2547 female) have been followed up prospectively since birth^(19,20)^. Medical, social, educational, and other data, including tests of functional capacity (from mid- to late life), anthropometry, and diet have been collected throughout life via home visits by research nurses, medical examinations during clinical visits, and postal questionnaires^(19,20)^. The cohort has been assessed on up to twenty-four occasions most recently in 2015 at the age of 69 years (for details see Supplementary Methods in Supplementary Material)^(21)^.

### Ethics

The MRC NSHD study was conducted according to the guidelines laid down in the Declaration of Helsinki. Relevant ethical approvals have been obtained for each data collection, with the most recent data collection at the age of 68–69 years approved by Queen Square Research Ethics Committee (13/LO/1073) and Scotland A Research Ethics Committee (14/SS/1009), and participants provided written informed consent. Written informed consent was obtained from each participant at each data collection. No additional ethical approvals were required for these secondary analyses of the MRC NSHD data.

### Dietary assessment

**Fig. 1.**
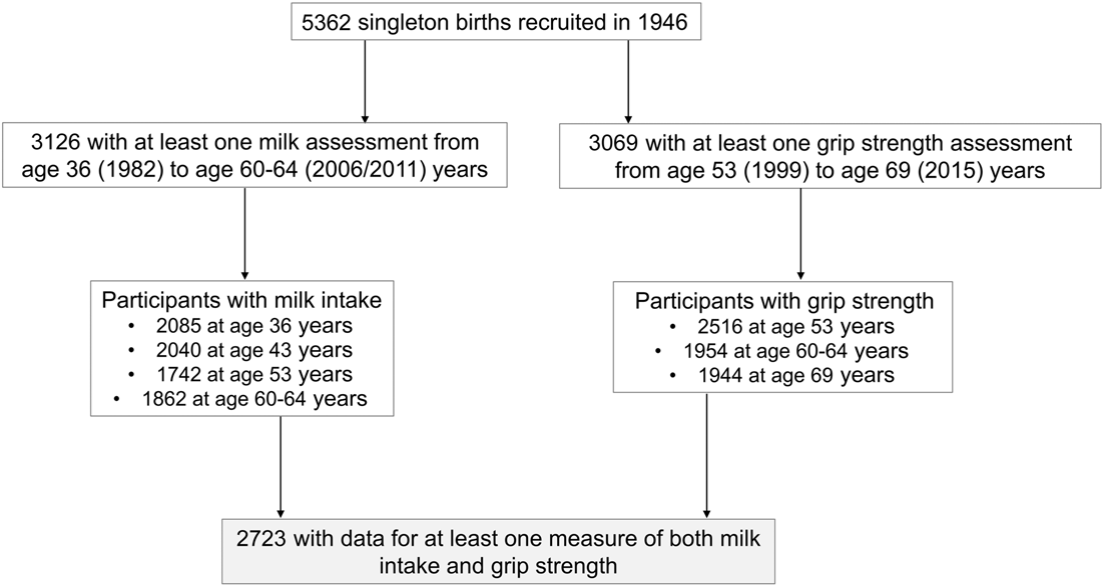
Flow chart of participants in the analytic sample. Of 5362 singleton births recruited to the MRC National Survey of Health and Development in 1946, 3126 participants had at least one milk assessment across adulthood (aged 36 to 60–64 years) and 3069 participants had at least one grip strength assessment from mid- to late life (aged 53 to 69 years). Of those, 2723 had data for at least one measure of both and comprised the analytic sample.

Details about dietary assessments for this cohort have been published previously^(22,23)^. Briefly, dietary data were collected using 5-d diet diaries on four occasions when participants were in the age of 36 (1982), 43 (1989), 53 (1999) and 60–64 years (2006–2011). All foods and drinks consumed, including animal milks by fat content and type (whole milk, semi-skimmed milk, 1 % fat milk, other animal milk except cow’s (e.g. goat) and flavoured milk (e.g. chocolate milk)), were coded by MRC Human Nutrition Research, Cambridge, with the in-house programmes based on McCance and Widdowson’s The Composition of Foods and its Supplements^(24)^, and gram per d (g/d) calculated. For the present study, dietary data of participants who completed at least 3 d of food diary at any one age were included, and intakes of each milk type at each age were calculated. A total of 3126 participants had at least one dietary assessment from 36 to 60–64 years of age (*n* 2085 at the age of 36 years, 2040 at the age of 43 years, 1742 at the age of 53 years and 1892 at the age of 60–64 years) (Fig. 1).

#### Milk intake

Only animal milks were used to calculate total milk, reduced-fat milk and full-fat milk intake (g/d) for each assessment from the age of 36 to 60–64 years. For reduced-fat milk, daily intakes of skimmed, semi-skimmed and 1 % fat milk were combined. For full-fat milk, daily intakes of whole milk, flavoured milk and other animal milk were combined. For total milk, reduced-fat and full-fat milk intake were combined for each assessment. For an average milk intake over approximately 28 years, grand means of intake for total, reduced-fat and full-fat milk were calculated (g/d; continuous) in 3126 participants, taking into account the number of dietary assessments each participant provided. All grand mean milk variables (total, full-fat and reduced-fat milk) had a positive skew so were each categorised in thirds (T1 (lowest) to T3 (highest) intake). Milk intake (g/d) by milk fat content in the analytic sample and by sex over approximately 28 years is presented in Supplementary Table 1 (Supplementary Material). Using the same approach, we also calculated grand means of total milk protein and total dietary protein intake (g/d), and their difference at each age to estimate the grand mean of protein intake from other foods (i.e. grand mean of non-milk protein) in the analytic sample and by sex from the age of 36 to 60–64 years (details in Supplementary Methods and Supplementary Table 1).

### Grip strength

GS (kg), a recommended measure of muscle strength for the assessment of probable sarcopenia by the European Working Group for Sarcopenia in Older People (EWGSOP2)^(12)^, was assessed by nurses following standardised measurement protocols at the age of 53, 60–64 and 69 years. At the age of 53 and 60–64 years, a Nottingham electronic handgrip dynamometer was used, and at the age of 69 years a Jamar Plus+ Digital Hand dynamometer was used^(25,26)^, and the measurements across the two devices were comparable^(27)^. For each GS assessment from the age of 53 to 69 years, we used the maximum of the first four measurements (two in left hand and two in right hand)^(26)^ to define the outcome at each age as recommended by established protocols for muscle strength (GS)^(28)^ if at least two measurements were available^(26)^. A total of 2516 (aged 53 years), 1954 (aged 60–64 years) and 1944 (aged 69 years) participants had valid GS measures at each age (Fig. 1).

We used sex-specific cut-offs for low GS (< 16 kg (women) and < 27 kg (men)) to define probable sarcopenia as recommended by the EWGSOP2 in 2019^(12)^. Maximum GS (kg) with absolute mean change for men and women and by sex-specific cut-offs (percentages) for low GS (probable sarcopenia) at each age are presented in Supplementary Table 2 (Supplementary Material).

### Analytic sample

Longitudinal data for both the exposure (milk) and outcome (GS) available for analyses from the age of 36 to 69 years are presented in Supplementary Fig. 1. The maximum sample (i.e. ‘analytic sample’) comprised 2723 participants (1340 (49·2 %) men and 1383 (50·8 %) women) with at least one exposure (milk intake from the age of 36 to 60–64 years) and one outcome (GS from the age of 53 to 69 years) (Fig. 1). Risk factors of muscle strength for the analytic sample were measured at midlife (aged 53 years), a period in the life course identified as a starting point for GS decline^(15)^. The age of 53 years was used as baseline for change in GS over time.

### Covariates

The following characteristics (variables) were included in univariable and multivariable analyses. Socio-demographic factors comprised sex, highest educational level attained (up to 26 years of age) and occupational class of the head of household (at the age of 53 years or at the age of 43 years if missing at 53 years of age). Health and lifestyle factors included health conditions, BMI (kg weight/m^2^ height, continuous), smoking status and leisure-time physical activity at the age of 53 years^(29)^. We also included a variable indicating attrition after the age of 53 years which distinguished those participants who were assessed later in life (at ages 60–64 and/or 69 years) from those participants assessed at the age of 53 years who did not participate at ages 60–64 and 69 years to account for losses to follow-up, and calculated intakes of total milk protein at each assessment and grand means of total milk protein and non-milk protein in the analytic sample and by sex over approximately 28 years (Supplementary Methods in Supplementary Materials; Supplementary Table 1).

### Statistical analysis

IBM SPSS v25 (SPSS, IBM Corporation) and R version 4.0.2 (R Foundation for Statistical Computing, Vienna, Austria; https://www.R-project.org/) were used for the analyses. The analytic sample was described using descriptive statistics across key socio-demographic, health and lifestyle variables. To compare men and women, we used a Student’s *t* test for normally distributed continuous variables, a Mann–Whitney *U* test for non-normally distributed and ordered variables, and a *χ* ^2^ test for categorical variables at *α* < 0·05 (Table 1). Data normality was determined by examining normality statistics (skewness, kurtosis, the Shapiro–Wilk test and Q-Q plots).

**Table 1.** Characteristics of the analytic sample* by sex at the age of 53 years (unless otherwise stated) (Numbers and percentages)

Characteristics†	Men		Women		*P* ‡
	*n*	%	*n*	%	
Grip strength (kg)					
Mean	47·7		28·08		< 0·001
sd	12·1		7·8		
Socio-demographic					
Sex	1340	49·2	1383	50·8	
Highest educational level attained (by the age of 26 years)					< 0·001
None	451	35·5	450	34·4	
Less than O-levels	74	5·8	120	9·2	
O-levels or equivalents	187	14·7	354	27·0	
A-levels or equivalents	360	28·3	311	23·7	
Degree or higher	200	15·7	75	5·7	
Occupational class§					0·008
High	133	10·1	186	13·7	
Intermediate	477	36·1	498	36·7	
Low	710	53·8	674	49·6	
	Mean	sd	Mean	sd	
Health					
Health conditions||	0·6	0·7	0·7	0·8	< 0·001
0	731	57·4	654	48·1	
1	398	31·2	500	37·3	
2	129	10·1	147	11·0	
≥ 3	16	1·3	50	3·7	
BMI (kg/m^2^)	27·3	3·9	27·4	5·4	0·002
	*n*	%	*n*	%	
Lifestyle					
Smoking status					
Current	269	21·1	283	21·1	
Never or former	1004	78·9	1059	78·9	
Physical activity¶					
None (inactive)	588	46·2	655	48·8	
1–4 times/month (intermediate)	238	18·7	229	17·1	
≥ 5 times/month (active)	446	35·1	458	34·1	
Grand mean of total milk					0·02
T1 (≤ 153·0 g/d)	421	47·8	460	52·2	
T2 (153·01–237·51 g/d)	434	46·8	494	53·2	
T3 (≥ 237·52 g/d)	485	53·1	429	46·9	
Grand mean of reduced-fat milk					< 0·001
T1 (≤ 58·75 g/d)	423	55·1	354	44·9	
T2 (58·76–145·25 g/d)	472	48·4	504	51·6	
T3 (≥ 145·26 g/d)	445	45·5	534	54·5	
Grand mean of full-fat milk					< 0·001
T1 (≤ 48·22 g/d)	416	43·6	538	56·4	
T2 (48·23–107·0 g/d)	461	47·9	501	52·1	
T3 (≥ 107·01 g/d)	463	57·4	344	42·6	
Attrition after the age of 53 years					0·005
Participated at the age of 60–64 and/or 69 years	1194	89·1	1276	92·3	
Did not participate at the age of 60–64 or 69 years	146	10·9	107	7·7	

MRC NSHD, Medical Research Council National Survey of Health and Development.

* MRC NSHD participants with at least one exposure (milk intake from the age of 36 to 60–64 years) and one outcome (grip strength from the age of 53 to 69 years).

† n varies because of missing data in some variables.

‡ Based on adjusted residuals for categorical variables.

§ Based on the head of household occupation (at the age of 53 years and, if missing, at the age of 43 years) and categorised using the Registrar General’s Social Classification.

|| Knee osteoarthritis and hand osteoarthritis, severe respiratory symptoms, and other disabling or life-threatening conditions.

¶ Any sports or vigorous leisure activities in the last month at the age of 53 years.

To examine the association between grand mean milk thirds (for total, reduced-fat and full-fat milk) and the risk of probable sarcopenia (sex-specific low GS) at the age of 69 years, we used logistic regression (OR 95 % CI) (Table 2). T1 (low milk intake) was used as a reference group in all models. Model 1 was unadjusted. Model 2 was adjusted for sex and occupational class; Model 3 (i.e. parsimonious model) was further adjusted for health and lifestyle variables (health conditions, BMI and leisure-time physical activity).

**Table 2.** The odds ratios of probable sarcopenia* at age 69 years by grand mean milk intake thirds in the analytic sample (Odd ratio and 95 % confidence intervals)

Grand mean of milk in thirds	Model 1		*P*	Model 2		*P*	Model 3		*P*
	OR	95 % CI		OR	95 % CI		OR	95 % CI	
*n*	1944			1924†			1854†		
Total milk, g/d									
T1 (≤ 153·00)	1			1			1		
T2 (153·01–237·51)	0·69	0·44, 1·08	0·1	0·69	0·44, 1·08	0·1	0·67	0·42, 1·07	0·1
T3 (≥ 237·52)	1	0·66, 1·5	0·99	0·99	0·65, 1·51	0·99	1·00	0·65, 1·55	0·99
Reduced-fat milk									
T1 (≤ 58·75)	1			1			1		
T2 (58·76–145·25)	0·59	0·38, 0·91	0·02	0·58	0·37, 0·90	0·02	0·59	0·37, 0·94	0·03
T3 (≥ 145·26)	0·68	0·44, 1·04	0·08	0·68	0·44, 1·05	0·08	0·69	0·44, 1·09	0·12
Full-fat milk									
T1 (≤ 48·22)	1			1			1		
T2 (48·23–107·00)	1·06	0·71, 1·59	0·77	1·07	0·71, 1·61	0·75	1·14	0·75, 1·73	0·55
T3 (≥ 107·01)	0·92	0·58, 1·44	0·71	0·90	0·57, 1·42	0·66	0·82	0·50, 1·33	0·42

Model 1 is unadjusted.

Model 2 is adjusted for socio-demographic variables (sex and occupational class).

Model 3 is further adjusted for health and lifestyle variables (health conditions, BMI, and leisure-time physical activity).

* Based on the sex-specific cut-offs for low grip strength (< 27 kg in men and < 16 kg in women)^(15)^. 1944 participants had grip strength data at the age of 69 years.

† Missing data for covariates: occupational class (*n* 20), health conditions (*n* 60), BMI (*n* 7) and leisure-time physical activity (*n* 3).

To investigate the associations between grand mean milk thirds and GS initial level (at the age of 53 years) and the rate of change over 16 years, we conducted multilevel linear modelling^(30)^ and fitted the following linear growth curve models in men and women in the analytic sample (Table 3). Model 1 was a ‘time’-only model and included age centred at 53 years (continuous (in years)) to test the linear trend of time. Model 2 included grand milk thirds of either total, reduced-fat or full-fat milk (to test whether initial status (intercept) varied by milk thirds), and an interaction term for milk and time to test for varying rates of change (slope) in GS by milk thirds. Model 3 was further adjusted for the same set of covariates as described above.

**Table 3.** *β* estimates of mixed models for muscle strength (grip strength) decline* from the age of 53 to 69 years in by grand mean milk intake† from mid- to late life in men and women in the analytic sample (Coefficients values and 95 % confidence intervals)

Exposure	Effect	Model 1§		*P*	Model 2||		*P*	Model 3¶		*P*
Men		*β* ‡	95 % CI		*β*	95 % CI		*β*	95 % CI	
Total milk	Time	–0·47	–0·51, −0·42	<0·001	–0·44	–0·52, −0·36	<0·001	–0·44	–0·52, −0·36	<0·001
g/d	T1 (≤ 153·0) (ref)				0			0		
	T2 (153·01–237·51)				1·30	–0·39, 2·99	0·13	1·07	–0·61, 2·75	0·21
	T3 (≥ 237·52)				1·54	–0·1, 3·18	0·06	1·82	0·18, 3·45	0·03
	Slope									
	T1 × Time (ref)				0			0		
	T2 × Time				–0·03	–0·14, 0·09	0·66	–0·02	–0·13, 0·09	0·72
	T3 × Time				–0·05	–0·16, 0·06	0·33	–0·06	–0·17, 0·05	0·29
Reduced-fat milk	Time	–0·47	–0·51, −0·42	<0·001	–0·43	–0·51, −0·34	<0·001	–0·44	–0·52, −0·35	<0·001
g/d	T1 (≤ 58·75) (ref)				0			0		
	T2 (58·76–145·25)				1·68	0·02, 3·33	0·05	1·25	–0·40, 2·9	0·14
	T3 (≥ 145·26)				1·44	–0·23, 3·11	0·09	1·23	–0·44, 2·89	0·15
	Slope									
	T1 × Time (ref)				0			0		
	T2 × Time				–0·07	–0·18, 0·05	0·25	–0·05	–0·17, 0·06	0·34
	T3 × Time				–0·05	–0·16, 0·07	0·40	–0·04	–0·16, 0·07	0·45
Full-fat milk	Time	–0·47	–0·51, −0·42	<0·001	–0·50	–0·58, −0·42	<0·001	–0·50	–0·58, −0·42	<0·001
g/d	T1 (≤ 48·22) (ref)				0			0		
	T2 (48·23–107·0)				–0·90	–2·57, 0·76	0·29	–1·02	–2·67, 0·64	0·23
	T3 (≥ 107·01)				0·90	–0·77, 2·57	0·29	1·21	–0·45, 2·87	0·15
	Slope									
	T1 × Time (ref)				0					
	T2 × Time				0·09	–0·02, 0·2	0·11	0·08	–0·03, 0·19	0·15
	T3 × Time				0·01	–0·1, 0·13	0·80	0·01	–0·10, 0·12	0·86
Women										
Total milk	Time	–0·25	–0·28, −0·22	<0·001	–0·24	–0·29, −0·19	<0·001	–0·25	–0·30, −0·20	<0·001
g/d	T1 (ref)				0			0		
	T2				0·55	–0·47, 1·57	0·29	0·24	–0·78, 1·27	0·65
	T3				–0·15	(0·54); −1·21, 0·91	0·78	–0·21	–1·27, 0·85	0·70
	Slope									
	T1 × Time (ref)				0			0		
	T2 × Time				–0·03	–0·10, 0·04	0·41	0·01	–0·06, 0·09	0·71
	T3 × Time				0·01	–0·06, 0·09	0·70	–0·03	–1·0, 0·05	0·48
Reduced-fat milk	Time	–0·25	–0·28, −0·22	<0·001	–0·31	–0·37, −0·24	<0·001	–0·31	–0·38, −0·25	<0·001
g/d	T1 (ref)				0			0		
	T2				–0·04	–1·15, 1·06	0·94	–0·26	–1·37, 0·86	0·65
	T3				–0·39	–1·49, 0·70	0·48	–0·45	–1·55, 0·66	0·43
	Slope									
	T1 × Time (ref)				0			0		
	T2 × Time				0·07	–0·01, 0·15	0·09	0·07	–0·01, 0·15	0·09
	T3 × Time				0·08	–0·0003, 0·16	0·051	0·08	–0·001, 0·16	0·053
Full-fat milk	Time	–0·25	–0·28, −0·22	<0·001	–0·24	–0·29, −0·19	<0·001	–0·24	–0·29, −0·20	<0·001
g/d	T1 (ref)				0			0		
	T2				0·29	–0·68, 1·26	0·56	0·28	–0·69, 1·25	0·57
	T3				–0·34	–1·43, 0·75	0·54	–0·26	–1·35, 0·84	0·65
	Slope									
	T1 × Time				0			0		
	T2 × Time				–0·02	–0·09, 0·05	0·52	–0·03	–0·10, 0·04	0·37
	T3 × Time				0·003	–0·07, 0·08	0·95	0·004	–0·07, 0·08	0·92

ref, Reference.

* Maximum grip strength measured four times in both hands for each assessment.

† Grand mean milk thirds of total, reduced-fat and full-fat milk from the age of 36 (1982) to 60–64 years (2006/2011) in participants who had complete data for milk intake (g/d) for at least 3 d of a 5-d diet diary at each diet assessment.

‡ Negative coefficients for grip strength indicate poorer performance.

§ Model 1 was unadjusted and included liner trend of time (age centred at 53 years), random intercepts and slopes.

|| Model 2 was further adjusted for grand mean milk thirds (total, reduced-fat and full-fat milk) and interaction terms (grand mean milk thirds × time).

¶ Model 3 was further adjusted for socio-demographic (occupational class), health and lifestyle variables (health conditions, BMI and leisure-time physical activity).

Further details about logistic and mixed models and rationale for the inclusion of covariates (i.e. known influences of GS) are described in Supplementary Methods (Supplementary Materials).

### Supplementary analysis

The analytic sample was further described by grand mean of total milk thirds and sex across key socio-demographic, health and lifestyle variables. For comparison, we used *χ* ^2^ tests for categorical variables, Kruskal–Wallis for ordinal and non-normally distributed variables, and one-way ANOVA for normally distributed, continuous variables *α* < 0·05 (online Supplementary Table 3). Multivariable supplementary analyses employed, and the results are presented in Supplementary Methods (Supplementary Methods and Supplementary Tables 4–10, respectively). We examined the robustness of the main findings using additional covariates, imputation and subsample analyses (online Supplementary Table 4–7). We also conducted a series of regression analyses to explore the cross-sectional associations between milk intake and GS (Supplementary Table 8 to Supplementary Table 10).

## Results

### Sample characteristics at the age of 53 years

The characteristics of participants in the analytic sample across a set of socio-demographic, health and lifestyle factors at the age of 53 years are presented in Table 1. Of 2723 participants in the sample (50·8 % women), over half belonged to low social class based on the occupation of the head of household, whilst 10·7 % had high educational attainment. Over 50 % had no health conditions (from the list), and 34·6 % reported engaging in leisure-time vigorous physical activity five or more times a month. Women were more likely to belong to lower occupational classes (*P* = 0·008) and to have contributed to data at the age of 60–64 and/or 69 years (*P* = 0·005) after 53 years of age, whilst men had stronger GS and higher levels of educational attainment (*P* < 0·001) and had fewer health conditions (*P* < 0·001) compared with women.

The characteristics of participants in the analytic sample by grand mean of total milk thirds and sex are reported in Supplementary Table 3. Briefly, participants in T3 (highest intake) were more likely to be men (*P* = 0·02) and to have higher educational attainment (*P* < 0·001), whilst participants in T1 (lowest intake) belonged to lower occupational class, were more likely to be current smokers, and had higher BMI (*P* < 0·001) compared with participants in T1 and T2. Women in T1 had lower occupational class (*P* = 0·001), were current smokers (*P* = 0·01) and had higher BMI (*P* = 0·002) compared with women in other milk thirds.

### Milk intake across adulthood in the analytic sample

Milk intake (total, reduced-fat and full-fat) across adulthood (from the age of 36 to 60–64 years) in all participants, men and women in the analytic sample is presented in Supplementary Table 1. Total milk intake was the lowest at the age of 36 years (mean (sd)) = 195·16 (119·63) g/d) and the highest at the age of 53 years (237·08 (141·0) g/d) and was in general higher in men than in women corresponding to higher milk protein intake in men at each age. Reduced-fat milk intake increased, and full-fat milk intake decreased over approximately 28 years, a trend reported previously in this cohort^(22)^. Grand means (sd) of total, reduced-fat and full-fat milk intake across adulthood were 205·6 (104), 121·6 (96·2) and 86·6 (79·4) g/d, respectively. Men were more likely to belong to T3 of both total (*P* = 0·02) and full-fat milk (*P* < 0·001) and to belong to T1 of reduced-fat milk (*P* < 0·001) compared with women (Table 1). In general, men had higher total protein and non-milk protein intakes compared with women (Supplementary Table 1).

### Muscle strength from the age of 53 to 69 years in the analytic sample

Supplementary Table 2 describes mean levels of GS and the prevalence of probable sarcopenia (i.e. low sex-specific GS) at each age in the analytic sample. GS declined in both men and women between the age of 53 and 69 years, an absolute change in mean maximum GS (sd) of −8·06 kg (11·53) in men, and −4·30 kg (7·95) in women. At the age of 69 years, the prevalence of probable sarcopenia was 6·9 % and was comparable in men and women and across other assessments (age of 53 and 60–64 years) in the analytic sample (all *P* ≥ 0·2; details not shown).

### Milk intake across adulthood and muscle strength from the age of 53 to 69 years

#### Milk intake and probable sarcopenia at the age of 69 years

Participants in T1 (≤ 58·75 g/d) of grand mean reduced-fat milk were more likely to have probable sarcopenia at the age of 69 years (*P* = 0·049) (details not shown). Table 2 presents the results from logistic regression models. In the parsimonious model (model 3), only T2 (58·76–145·25 g/d) of grand mean reduced-fat milk was associated with lower odds of probable sarcopenia at the age of 69 years (OR (95 % CI): 0·59 (0·37, 0·94), *P* = 0·03) compared with T1, after adjusting for key covariates at midlife (sex, occupational class, health conditions, BMI and leisure-time physical activity). The odds for T2 remained lower (0·60 (0·37, 0·98), *P* = 0·04) after further adjustment for education, smoking status and attrition after the age of 53 years in the saturated models and after further adjustment for grand mean intakes of protein from other foods (non-milk protein) (online Supplementary Table 4). No associations with other types of milk (grand mean of total and full-fat thirds) were observed (Table 2).

#### Milk intake and change in grip strength over time

**Fig. 2.**
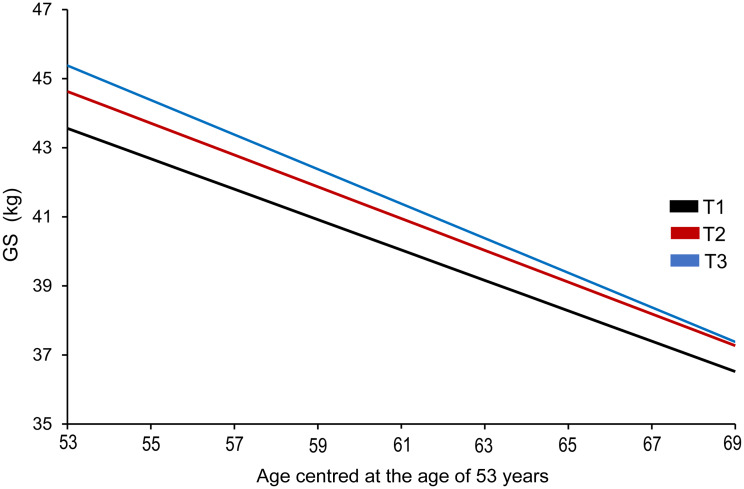
Estimated 16-year trajectory of grip strength by grand mean of total milk thirds across adulthood in men. In the model adjusted for key covariates (model 3), only the highest intake of grand mean of total milk was associated with grip strength (GS) in midlife in men, but not with GS decline over 16 years. Although men in T3 (≥ 237·52 g/d; blue line) had higher GS (kg) in midlife (aged 53 years) compared with those in T1 (≤ 153·0 g/d; black line), no differences in the rate of decline in GS across the milk thirds were observed over time. GS, grip strength.

Table 3 presents estimates from mixed effect models for GS change (kg) over 16 years in men and women, and GS trajectories by grand mean milk thirds for significant results were plotted graphically (Fig. 2, online Supplementary Fig. 2).

In model 3, which included the fixed effect of time (age centred at 53 years; midlife), grand mean milk thirds (T1 (lowest) and T3 (highest) intake, g/d), milk × time interaction term and key covariates (occupational class, health conditions, BMI and physical activity), T3 of total milk (≥ 237·52 g/d) was associated with higher GS in midlife (*β* (95 % CI) = 1·82 (0·18, 3·45)) in men compared with T1 (≤ 153·0 g/d). Trajectory of GS by grand mean total milk thirds (model 3) in men over 16 years (i.e. between 53 and 69 years of age) is presented in Fig. 2, showing an overall decline over time, but no difference in the rate of change across the milk thirds. However, these associations were not robust to additional adjustments for education, smoking status, attrition after the age of 53 years (saturated model 1: 1·66 (0·01, 3·33), *P* = 0·052) and grand mean of non-milk protein (saturated model 2: 1·49 (–0·20, 3·17), *P* = 0·08) (online Supplementary Table 6).

No fixed effects of grand mean milk thirds (total milk, reduced-fat milk and full-fat milk) on GS were found in women, and GS slopes did not vary by grand mean milk thirds in any of the parsimonious models (model 3; Table 3), except for T3 of reduced-fat milk in the saturated model (online Supplementary Table 6), showing a slower rate of GS decline compared with T1 (online Supplementary Fig. 2). Because of large CI, the result should be interpreted with caution.

### Supplementary analysis

#### Multivariate analyses with imputed covariates and in complete covariates subsample

For imputed analyses, similar results were observed in logistic models (parsimonious and saturated) showing 40 % decreased odds of probable sarcopenia being associated with T2 (58·76–145·25 g/d) of reduced-fat milk in the analytic sample (online Supplementary Table 4). The results were also very similar for mixed linear models compared with the main analyses. T3 of total milk was associated with higher GS at baseline (aged 53 years) in men in both the parsimonious (model 3) and saturated models, but not with GS decline over time (online Supplementary Table 7). For women, T3 of reduced-fat milk showed a slower rate of GS decline in both the parsimonious and saturated model (online Supplementary Table 7). Large CI preclude the certainty of the estimates. Over 89 % of participants (*n* 2445) in the analytic sample had complete data for covariates. Both OR and *β* estimates in the subsample were comparable to those in the main and imputed analyses (Supplementary Table 5 and 7, respectively).

#### Linear regression models for milk intake and grip strength

In multivariable linear regression models with milk intake transformed (√x), we observed the following statistically significant effects sizes (*β* coefficients) for GS (kg). Total milk intake at the age of 60–64 years was associated with higher GS at the same age (a 0·09 kg per unit increase in total milk (√g/d), *P* = 0·04) in the saturated model (model 3; Supplementary Table 8). Similar associations were found for reduced-fat milk intake after adjustment for key covariates (model 2). Total milk intake at the age of 36 years (model 2: *β* = 0·04, *P* = 0·04), 43 (*β* = 0·02, *P* = 0·02), and 60–64 years (*β* = 0·07, *P* = 0·03) was positively associated with GS at the age of 69 years. Significant positive associations were also observed with full-fat milk intake at the age of 43 years (*β* = 0·07, *P* = 0·02) and reduced-fat milk at the age of 60–64 years (*β* = 0·08, *P* = 0·009) and GS at the age of 69 years (online Supplementary Table 9). However, the grand means of milk intake across adulthood (total, reduced-fat and full-fat) were not associated with GS in late life in the adjusted models (model 2 and 3; online Supplementary Table 10).

## Discussion

### Summary of results

We investigated the relationship between milk intake across adulthood (from the age of 36 to 60–64 years) and GS from mid- (aged 53 years) to late life (aged 69 years) in 2723 participants from the MRC NSHD. In multivariate analyses, higher grand mean (≥ 237·5 g/d) of total milk intake was associated with higher GS in midlife in men, and this was maintained after adjustment for key covariates including known influences on muscle strength (including sex, occupational class, BMI, health conditions and physical activity)^(25,26)^ (online Supplementary material S1–S5), but not with GS decline over 16 years. Additionally, higher total milk intake at ages 36, 43 and 60–64 years was associated with stronger GS in late life (aged 69 years). For reduced-fat milk, T2 of grand mean (58·8–145·2 g/d) was associated with a 40 % lower risk of probable sarcopenia at the age of 69 years after including key covariates in all participants and was robust to additional adjustments, including protein intake from other food sources. No clear associations were found in women.

### Comparison with existing literature and interpretation of results

To our knowledge, this is one of a few prospective studies investigating the link between milk intake (at any point in life) and muscle-related outcomes (mass, strength, physical performance and sarcopenia) in late life, thus making the comparison of the findings across studies challenging. Our recent narrative synthesis of epidemiological evidence revealed only three prospective studies with both milk intake and muscle health parameters assessed only once throughout the life course^(7)^. The studies were heterogenous and differed greatly by the period in life when milk intake was measured (from childhood to late life) and how muscle health was determined in late life. Validated FFQ were the commonest way to estimate milk intakes (total or by fat content), which, because of skewed distributions, were expressed in medians (g/d) or categories (e.g. quartiles of intake, pints in categories and per diet score category). Briefly, in the Boyd Orr study of 405 older adults (aged ≥ 70 years), no associations were found between total milk intake (type not specified) in midlife (none, < ½ pint, ½ to 1 pint, > 1 pint) and walking times, and higher milk intake was associated with poorer balance in late life^(31)^, whilst a unit increase (½ pint milk/d) of full-fat milk at the age of 59–73 years was associated with 21 % lower risk of poor balance at the age of 66–86 years in > 1000 men in the Caerphilly Prospective Study^(31)^. In the Helsinki Birth Cohort Study of over 1000 adults aged ≥ 60 years at baseline, low consumption of reduced-fat milk (< 2 % fat and fat-free in sex-specific quartiles of average daily intakes (g/d) and as a part of a healthy Nordic diet score (NDS)) was independently associated with better physical performance assessed by the Senior Fitness Test (including chair stands and arm curls) at 10-year follow-up in men, but not in women^(32)^. However, a separate study from this cohort has shown that GS was 5 % and leg extension strength 7 % greater in women (but not in men) who belonged to the highest quartile of the NDS score compared with those in the lowest quartile, but milk was not the component that contributed to these association^(33)^.

Taken together, the results regarding the amount and type of milk (by fat content) and muscle health from mid- to late life from the aforementioned observational studies were inconclusive. Similarly, our results on the relationships between milk (by types and amounts in thirds) and GS in men and women from mid- to late adulthood were mixed. We have found some evidence for lower risk of probable sarcopenia in later life in participants consuming 59–145 g/d of reduced-fat milk across adulthood (aged 36 to 60/64 years) and not in those with higher intake. However, no clear dose–response was observed, and the results need to be interpreted with caution. Associations for full-fat milk were not consistent, and the total milk intake was associated with stronger GS initially (aged 53 years) but not over time in men and not in women. Although we adjusted for occupational (social) class, physical activity and disease burden, the latter could be explained by marked sex differences in occupations and types of physical activities undertaken by men compared with women which may benefit muscle strength and by uncontrolled confounding (e.g. differences in change in key covariates over time). Experimental evidence in young adults has shown that milk with higher content of milk fats might be beneficial for muscles by aiding better absorption of essential amino acids for muscle anabolism after resistance exercise^(34)^. Although full-fat milk may be relatively easy to incorporate into a balanced diet of an older person as a nutritious and affordable whole food, little is known about how milk consumption in amounts providing > 20 g of protein/d needed to overcome anabolic resistance^(35)^ may affect muscle health in late life.

Regarding reduced-fat milk intake, the results from the Helsinki cohort of older adults were mixed; only lower consumption (approximately 137 g/d in the lowest quartile of the NDS and not amounts > 226 g/d in higher NDS quartiles) in early late life (≥ 60 years) was associated with better fitness score (including muscle strength) in men a decade later, but reduced-fat milk was not a component of the NDS contributing to greater GS and leg extension strength in older women^(32)^. We have found some positive associations between reduced-fat milk and GS, which need to be interpreted with caution as specified below. Along with T2 (59–145 g/d) of reduced-fat milk being associated with 40 % lower odds of probable sarcopenia (sex-specific low GS), other significant associations were found with higher intake at the age of 60–64 years and greater GS at the same age and at the age of 69 years. However, the fixed effects estimates for women belonging to T3 of reduced-fat milk (> 145·3 g/d) experiencing slower rate of GS decline compared with those in the lowest third (saturated and imputed models; Supplementary Fig. 2) were uncertain although being robust to additional adjustments (including attrition and non-milk protein intake). Also, the non-linear association observed in reduced-fat milk and probable sarcopenia relationship (i.e. T2 of milk intake significantly associated but T3 not) may have resulted from residual confounding, bias sample or spurious findings.

Aside from observational studies synthesised in our previous narrative review^(7)^, we have found only one additional study that investigated the association between total milk intake frequency (from FFQ and 24-h recall), muscle mass and GS in Korean adults aged 19 to 69 years belonging to three population-based cohorts, including the 2008–2011 and the 2014–2016 Korean National Health and Nutrition Examination Survey (KNHANES)^(36)^. Milk intake frequencies were dichotomised to < 1 a day or ≥ 1 a day from ten categories (from rarely to two to three times a month to three times a day), but no amounts (servings) were estimated (per the Korean Dietary Reference Intake, one serving of milk/dairy product is the amount of food providing 125 kcal). In the 2014–2016 KNHANES in over 13 500 individuals, those in the ≥ 1 a day milk group had on average 0·8 kg greater GS compared with those in < 1 a day group after multiple adjustments, including age, sex, lifestyle and number of chronic diseases. We have found some evidence for the associations with total milk intake defined as either grand mean (habitual intake across adulthood) or milk intake (g/d) at each time point over approximately 28 years. For grand mean in thirds, men consuming ≥ 237·5 g/d (T3) had higher GS in midlife compared with those in T1 (≤ 153 g/d) in the model adjusted for known influences of GS (e.g. health conditions, BMI and physical activity)^(25,26)^ (online Supplementary material S5) and other cohorts of middle-aged and older adults (online Supplementary material S1–S4). However, the results were not robust to additional adjustments (education, attrition, smoking and non-milk protein intake), and these covariates were not independently associated with GS (details not shown). No associations were found for GS change over 16 years (from mid- to late life) or in women. Higher total milk intake at the age of 60–64 years was positively associated with GS at the same age and at the age of 69 years.

Taken together, it could be postulated that habitual intake of milk across the life course providing an exceptional mixture of nutrients, including fast-acting soluble proteins – easily delivered in a liquid form and essential for muscle anabolism – may represent an important part of a balanced diet for muscle health in older adults. However, the lack of prospective studies for pooled analysis and the heterogeneity of the existing ones makes any assumptions about the strength of this association challenging.

### Relevance of results

Here we used longitudinal data from the 1946 British Birth Cohort and a broad life course approach^(37)^ to study the relationship between milk intake and muscle strength. Life course epidemiology of health and disease risk posits that numerous biological and social factors throughout the life course influence health and disease trajectory in adult life by acting either independently, cumulatively or interactively^(37)^. We used milk as a cumulative exposure assessed over approximately 28 years during the period in life characterised by muscle strength peak (early 30s), followed by plateau (40s) and decline (50s and later) and linked it to a specific time window of progressive change in skeletal muscle with ageing (through 50s to late 60s). The accumulation of exposure (milk) may result in long-term changes in biological systems (muscle) and exert either positive or negative influence on the system as the duration, type and intensity of exposure changes (e.g. total *v*. reduced-fat *v*. full-fat milk).

Using data from the birth cohort, both time in terms of lifetime (i.e. chronological age of individuals) for examining GS trajectories and historic time/period at the population level (as indexed by the birth cohort membership) become important in understanding the association between milk and muscle strength. Changes in the milk-type preference over approximately 28 years in this cohort have been described previously, with full-fat milk being the most commonly consumed type at the age of 36 years and declining thereafter and reduced-fat milk intake increasing over time^(22)^. Because of the prospective nature of the study, it is hard to disentangle the age effect from the time/period effect, and in the case of preferring reduced-fat milk over full-fat milk, this change could be explained by both availability of the new food (milks with low fat content) and dietary recommendations favouring low-fat diet. With current growth in the consumption of plant-based milks^(38)^, it should be acknowledged that the time/period effect on food choices and consumption of new milk alternatives in the later cohorts of older adults might be greater.

Changes in nutrient intake from the age of 36 to 53 years have been also described in this cohort (e.g. decrease of fat and K, but increase in Ca, folic acid, and vitamins C, D, and E) and explained by change in the consumption of several key whole foods (e.g. increased consumption of fruits and vegetables and decreased consumption of full-fat milk, butter and red meats), which were socio-economically patterned^(24)^. These changes need to be considered when interpreting the associations reported in this study. Proposed beneficial associations between reduced-fat milk and muscle strength could be confounded by or contributed to a simultaneous increase in other healthy foods and reduction of those negatively associated with overall health.

We have chosen midlife as a potentially important period in the life course postulating that midlife factors may exert strong effects on muscle health with long-lasting consequences in late life. The importance of midlife, especially for modifiable lifestyle factors influencing health and disease development in late life^(39)^, including those of muscle^(16,17,40)^, has been recognised in a number of studies.

### Strength and weaknesses

This study has several strengths, including (a) longitudinal design of the NSHD with long-term follow-up allowing for a life course approach; (b) repeated measures for muscle strength (GS) and diet from mid- to late life, including different types of milk for more complex analyses between exposure and outcome); (c) prospective ascertainment of a number of potential confounders and inclusion of known influences of GS; (d) representativeness of the population born in England, Scotland and Wales in the late 1940s^(41)^; and (e) robust analyses of change in GS (e.g. within- and between-person change) in the maximised sample to increase power. However, several weaknesses should be considered when interpreting the results, including (a) bias introduced by attrition and higher rates of mortality being previously reported in participants with low GS at the age of 53 years over 13 years of follow-up^(42)^; (b) higher health consciousness in those completing diet diaries^(24)^; (c) spurious findings because of a large number of associations tested; (d) residual confounding such as change in key covariates (occupational class, BMI, physical activity and health conditions) from mid- to late life, and uncontrolled confounding (e.g. change in daily activities and quality of life close to retirement) possibly contributing to inconsistent results across milk types and sexes; and (e) only linear trends being explored because of three data points being available for GS change.

### Conclusions

We aimed to investigate the associations between milk intake (total, reduced-fat and full-fat) across adulthood and change in muscle strength (GS) in mid- to late life using a birth cohort with long-term follow-up. We found some evidence to suggest that a higher total milk intake was associated with greater muscle strength in midlife (aged 53 years) in men, and higher reduced-fat milk intake predicted lower odds of probable sarcopenia (sex-specific low GS) in later life (aged 69 years) compared with low intake in all participants. Milk intake was not associated with change in muscle strength over time, and no clear associations were found in women. The results need to be corroborated in other birth cohorts of ageing born in different decades and from other countries to understand the nutritional value of milk for muscle health in late life and to aid future causal inference work.

## Supplementary material

For supplementary material accompanying this paper visit https://doi.org/10.1017/S0007114522001799. click here to view supplementary material
