# Supplementary material for: Milk intake across adulthood and muscle strength decline from mid- to late life: the MRC National Survey of Health and Development
Source: Br J Nutr. 2022 Jul 7;129(5):820–31. doi: 10.1017/S0007114522001799 (PMC9975781; doi:10.1017/S0007114522001799)
Supplement: Supplementary file 1 [file S0007114522001799sup001.docx]

**Supplementary Material**

**I. Supplementary Methods**

**II. Supplementary univariable analyses**

**Supplementary Table 1.** Milk, milk protein, total protein, and non-milk protein intake by sex from age 36 to 60-64 years in the analytic sample

**Supplementary Table 2.** Maximum grip strength by sex and the prevalence of probable sarcopenia from age 53 to 69 years in the analytic sample

**Supplementary Table 3**. Characteristics of the analytic sample (n=2723) at age 53 years by grand mean of total milk thirds and sex

**III. Supplementary multivariable analyses**

**Supplementary Table 4**. The risk of probable sarcopenia at age 69 years by grand mean milk intake thirds in the analytic sample: saturated models

**Supplementary Table 5.** The risk of probable sarcopenia at age 69 years by grand mean milk intake thirds in the analytic sample: parsimonious and saturated model with imputed covariates and in the subsample with complete covariates

**Supplementary Table 6**. β estimates of mixed models for muscle strength (grip strength) decline from age 53 to 69 years by grand mean milk intake from mid- to late life in men and women in the analytic sample: saturated models

**Supplementary Table 7.** β estimates of mixed models for muscle strength (grip strength) decline from age 53 to 69 years by grand mean milk intake from mid- to late life in men and women in the analytic sample: imputed analyses and in the subsample with complete covariates

**Supplementary Table 8**. Linear regression summary of effect sizes for milk intake predicting contemporaneous grip strength (kg) in the analytic sample

**Supplementary Table 9**. Linear regression summary of effect sizes for milk intake at ages 36, 43, 53 and 60-64 years predicting grip strength (kg) at age 69 years in the analytic sample

**Supplementary Table 10**. Linear regression summary of effect sizes for grand mean milk intake predicting grip strength (kg) at age 69 years in the analytic sample

**IV. Supplementary Figures**

**Supplementary Figure 1.** Timeline of exposure (milk) and outcome (grip strength) in the MRC National Survey of Health and Development

**Supplementary Figure 2** Estimated 16-year trajectory of grip strength by grand mean reduced-fat milk thirds across adulthood in women in the analytic sample

**V. Supplementary References**

**I.** **Supplementary Methods**

***Study population***

The 24^th^ data collection was conducted between 2014 and 2015 when study members were aged 68-69 years^(21)^. At age 69, following a postal questionnaire at age 68, study members still alive and with a known current address in mainland Britain (n = 2698) were invited to have a home visit; 2149 (79.7%) completed a visit and a further 55 (2.0%) completed a postal questionnaire instead. Of the original cohort, 1026 (19.1%) had died, 578 (10.8%) were living abroad, 22 (0.4%) asked for their participation to be restricted to postal contacts, 621 (11.6%) had previously withdrawn from the study, and 417 (7.8%) had been lost to follow-up^(21)^.

***Description of covariates***

Socio-demographic variables (sex, education, and occupational class): Highest educational level attained was categorised into: no qualifications, less than O-level (vocational course, and less than ordinary secondary education), ‘O-level’ or equivalents (ordinary secondary), ‘A-level’ or equivalents (advanced secondary education), degree or higher (masters, doctorate, or graduate equivalent)^(25,26)^. Occupational class was categorised as high (I professional and II intermediate occupations), intermediate (III Non-manual skilled and III Manual skilled occupations), and low (IV partly skilled and V unskilled occupations) based on the Registrar General’s Social Classification^(25,26)^.

Health and lifestyle variables (health conditions, BMI, leisure-time physical activity, and smoking status): Health conditions were a summary score (0-4, and categorised into 0, 1, 2, ≥3) of the following conditions: clinically diagnosed knee osteoarthritis and hand osteoarthritis, severe respiratory symptoms, and other disabling or life-threatening conditions^(28)^. Smoking status was categorised into current, and former or never smoker. For leisure-time physical activity, participants were asked how many times they had engaged in any sports or vigorous leisure activities in the last month (categorised into none, 1-4 times/month, ≥5 times/month).

Protein intake (protein in total milk and total dietary protein; g/day): The amount of protein in total milk intake in all participants and by sex from age 36 (1982) to 60-64 years (2006/2011) in the analytic sample was calculated in those who had complete data for milk intake (g/day) for at least 3 days of a 5-day diet diary at each assessment, and it was based on McCance and Widdowson's The Composition of Foods and its Supplements^(24)^. Similarly, total dietary protein intake was estimated at each assessment in all participants with at least 3 days diaries, and grand mean of protein intake across four measurements calculated in the analytic sample. From those, grand mean of non-milk protein intake was calculated by subtracting grand mean of total milk protein from grand mean of total (dietary) protein intake to examine whether protein from other foods influence the milk and grip strength relationship in multivariate analyses.

***Main statistical analyses***

*Logistic regression models.* We used a parsimonious approach with the covariates included and considered those previously shown to be associated with GS in this cohort^(25,26,S5)^, in the literature^(S1-S4)^ and those that contributed significantly to the model fit (e.g., a significant change in -2 log likelihood) to avoid over-adjustment and preserve the number of cases in the model. All covariates had <5% of missing data (e.g., 1.7% (occupational class), 3.9% (health conditions), 4% physical activity in the analytic sample; details not shown), thus a single imputation was performed in the supplementary analyses.

*Linear mixed modes*. Linear mixed models allow simultaneous examination of individual trajectory of change (within-person variability or random effects at Level 1), and population averages (between-person variability or fixed effects at Level 2) by using all available measurement and including participants with incomplete data. At Level 1, the models evaluate how GS changes over ~16 years for each participant, and at Level 2 the models determine the effect of covariates on GS (initially (at age 53 yeas) and over time).

Similar to logistic regression models, in Table 3 we reported parsimonious models with minimal number of parameters, and based on the model fit statistics (e.g., Akaike Information Criterion, AIC), whilst further adjustments were conducted in supplementary analyses. Following the simultaneous inclusion of all main effects, plausible interactions were tested (e.g., sex × time to test sex differences in rate of change in GS over time) in the analytic sample (n=2723). Men and women were analysed separately because of statistically significant differences in unadjusted GS between the sexes, and a significant sex × time interaction term observed in the initial mixed models during the model building strategy in all participants (e.g., sex × time: β (95% CI) = 0.21 (0.16, 0.27), p<0.001).

Random effects included random slopes and intercepts. Negative (declining) β estimates for GS indicated poor muscle strength (in kg). The SPSS MIXED procedure with maximum likelihood method and a scaled identity matrix (id) at Level 1 (within-subject change), and unstructured (UN) covariance matrix at Level 2 (between-person change) were used to generate parameter estimates (β) for effects.

***Supplementary analyses***

We conducted the following supplementary multivariable analyses (Supplementary Table 4 to Supplementary Table 10). To examine the robustness of the findings reported from the main analyses we used three approaches. First, both logistic regression and multilevel linear models (Model 3) were additionally adjusted for education (at age 26 years), smoking status, and attrition after age 53 years (i.e., Saturated Models 1 in Supplementary Table 4 and Supplementary Table 6, respectively). Furthermore, to examine the influence of other protein-rich foods on the milk and grip strength relationship, the models were additionally adjusted for grand mean of non-milk protein intake (Saturated Model 2 in Supplementary Table 4 and Supplementary Table 6).

Parsimonious and Saturated Models 1 were further repeated with the imputed covariates (mean for continuous (BMI, health conditions) and reference value for categorical variables (occupational class, leisure-time physical activity, highest educational level attained, smoking status), and also in the subsample comprising of participants with complete data for covariates (n = 2445 (89.8% of the analytic sample)) (Supplementary Table 5 and Supplementary Table 7, respectively).

Furthermore, we conducted a series of linear regression models to explore the following associations in the analytic sample: (a) milk intake (total, reduced-fat, full-fat) at age 53 and 60-64 years and GS at each corresponding age (Supplementary Table 8); (b) milk intake (total, reduced-fat, and full-fat) at ages 36, 43, 53, and 60-64 years and GS at age 69 years (Supplementary Table 9); (c) grand mean milk intake (total, reduced-fat, and full-fat) and GS at age 69 years (Supplementary Table 10). Positive skew for each milk type (total, reduced-fat, and full-fat) at each measurement occasion was corrected as √x (x representing original value), which improved the distribution for most variables based on the skewness and kurtosis statistics and Q-Q-plots, and allowed for 0 g/day values (i.e., non-consumption) to be retained in the calculations. Models were adjusted for the same set of covariates as in the main logistic regression and linear multilevel models (Supplementary Table 8 to Supplementary Table 10).

**II. Supplementary univariable analyses**

Supplementary Table 1. Milk^a^, milk protein^b^, total protein^c^, and non-milk protein^d^ intake by sex from age 36 to 60-64 years in the analytic sample

| **Age**  **(year of assessment)** | **Total milk**  **Milk protein** | **Reduced-fat milk^e^** | **Full-fat milk^f^** | **Grand mean of total milk**  **Grand mean of milk protein**  **Grand mean of non-milk protein** | **Grand mean of reduced-fat milk** | **Grand mean of**  **full-fat milk** | **Total protein intake** |
| --- | --- | --- | --- | --- | --- | --- | --- |
|  | M (SD) g/day | M (SD), g/day | M (SD) g/day | M (SD) g/day | M (SD) g/day | M (SD) g/day | M (SD) g/day |
| **36 (1982), n** | 2085 | 2085 | 2085 |  |  |  | 2080 |
| all | 195.16 (119.63)  6.83 (4.19) | 9.45 (47.77) | 185.72 (119.99) | - | - | - | 68.88 (20.96) |
| men | 204.46 (123.15)  7.16 (4.31) | 4.93 (35.39) | 199.53 (122.80) | - | - | - | 78.46 (21.33) |
| women | 186.38 (115.58)  6.52 (4.05) | 13.71 (56.74) | 172.66 (115.83) | - | - | - | 59.84 (16.01) |
| **43 (1989), n** | 2040 | 2040 | 2040 |  |  |  | 2042 |
| all | 220.73 (124.48)  7.73 (4.36) | 133.03 (144.15) | 87.71 (118.65) | - | - | - | 76.39 (21.66) |
| men | 226.42 (128.05)  7.93 (4.48) | 120.48 (142.38) | 105.95 (130.81) | - | - | - | 85.46 (22.13) |
| women | 215.31 (120.79)  7.54 (4.23) | 145.0 (144.88) | 70.31 (102.84) | - | - | - | 67.74 (17.21) |
| **53 (1999), n** | 1742 | 1742 | 1742 |  |  |  | 1742 |
| all | 237.08 (141.0)  8.30 (4.94) | 213.56 (148.27) | 23.52 (70.55) | - | - | - | 77.46 (18.25) |
| men | 237.39 (145.12)  8.31 (5.08) | 212.34 (152.0) | 25.06 (75.05) | - | - | - | 85.57 (18.68) |
| women | 236.81 (137.40)  8.29 (4.81) | 214.62 (145.02) | 22.19 (66.41) | - | - | - | 70.42 (14.58) |
| **60-64 (2006/11)** | 1862 | 1862 | 1862 |  |  |  | 1862 |
| all | 193.65 (132.36)  6.78 (4.63) | 174.72 (130.07) | 18.92 (72.95) | - | - | - | 76.46 (18.26) |
| men | 200.78 (138.81)  7.03 (4.86) | 177.50 (135.87) | 23.27 (85.12) | - | - | - | 83.30 (18.85) |
| women | 187.28 (126.05)  6.56 (4.41) | 172.24 (124.70) | 15.04 (59.83) | - | - | - | 70.14 (15.13) |
| **36 to 60-64^g^, n** |  |  |  | 2723 | 2723 | 2723 | 2721 |
| all |  | - | - | 205.55 (103.98)  7.19 (3.64)  45.97 (23.24) | 121.59 (96.17) | 86.59 (79.41) | 52.96 (24.07) |
| men |  | - | - | 210.81 (107.97)  7.38 (3.78)  50.05 (25.11) | 115.27 (96.16) | 96.82 (87.78) | 57.29 (25.90) |
| women |  | - | - | 200.46 (99.73)  7.02 (3.49)  41.97 (20.50) | 127.71 (95.81) | 76.68 (68.95) | 48.76 (21.34) |

^a^ Milk intake from age 36 (1982) to age 60-64 (2006/2011) years in participants who had complete data for milk intake (g/day) for at least 3 days of a 5-day diet diary at each diet assessment.

^b^ The amount of milk protein in total milk consumed from age 36 (1982) to age 60-64 (2006/2011) years in participants who had complete data for milk intake (g/day) for at least 3 days of a 5-day diet diary at each assessment was estimated based on McCance and Widdowson's The Composition of Foods and its Supplements^(24)^. From those, grand mean of milk protein intake was calculated over ~28 years (from age 36 to 60-64 years).

^c^Total protein intake was estimated from all foods based on McCance and Widdowson's The Composition of Foods and its Supplements^(24)^ at each assessment in participants with at least 3 days of a 5-day diet diary, and grand mean of total protein intake from age 36 (1982) to age 60-64 (2006-2011) years calculated.

^d^The amount of non-milk protein intake was calculated by subtracting grand mean of milk protein intake from grand mean of total protein intake in the analytic sample.

^e^Daily intakes of skimmed, semi-skimmed, and 1% fat milk combined.

^f^Daily intakes of whole milk, flavoured milk (e.g., chocolate milk), and other animal milks (e.g., goat) combined.

^g^Mean intake of four assessments over ~28 years (from age 36 to 60-64 years).

Supplementary Table 2. Maximum grip strength by sex and the prevalence of probable sarcopenia^a^ from age 53 to 69 years in the analytic sample

| **GS assessment** | **Men** | **Women** | **Normal GS in all participants** | **Low GS in all participants** | **p**^b^ |
| --- | --- | --- | --- | --- | --- |
|  | M (SD), kg | M (SD), kg | n (%) | n (%) |  |
| **age 53 (1999)** | 47.66 (12.06) | 28.08 (7.79) | 2379 (94.6) | 137 (5.41) | <0.001 |
| **age 60-64 (2006/11)** | 44.70 (11.72) | 26.04 (7.36) | 1813 (92.8) | 141 (7.2) | <0.001 |
| **age 69 (2015)** | 40.22 (8.43) | 24.17 (5.74) | 1809 (93.1) | 135 (6.9) | <0.001 |
| **Δ absolute** | -8.06 (11.53) | -4.30 (7.95) |  |  |  |

GS, grip strength

^a^Based on the sex-specific cut-offs for low grip strength (<27 kg in men, and <16 kg in women).

^b^χ^2^ test.

Supplementary Table 3. Characteristics of the analytic sample (n=2723) by grand mean of total milk thirds and sex at age 53

|  |  | **T1 of grand mean total milk (≤153.0g/day)** | | | **T2 of grand mean total milk**  **(153.01-237.51g/day)** | | | **T3 of grand mean total milk**  **(≥237.52g/day)** | | |  |  |  |
| --- | --- | --- | --- | --- | --- | --- | --- | --- | --- | --- | --- | --- | --- |
| **Characteristics** | **Analytic sample** | **All** | **Men** | **Women** | **All** | **Men** | **Women** | **All** | **Men** | **Women** | **p*^analytic sample^*** | **p*^men^*** | **p*^women^*** |
| *Outcome* |  |  |  |  |  |  |  |  |  |  |  |  |  |
| Grip strength (kg), M (SD) |  | 36.98 (13.60) | 46.92 (11.72) | 27.22 (7.51) | 37.55 (13.86) | 47.91 (11.71) | 28.52 (8.04) | 38.57 (14.72) | 48.06 (12.64) | 27.72  (7.80) | 0.07 | 0.35 | 0.29 |
| *Socio-demographic* |  |  |  |  |  |  |  |  |  |  |  |  |  |
| Sex, n (%) |  |  |  |  |  |  |  |  |  |  | 0.02 | - | - |
| Men | 1340 (49.2) | 421 (47.8) | - | - |  | - | - |  | - | - | - | - | - |
| Women | 1383 (50.8) | 460 (52.2) | - | - |  | - | - |  | - | - | - | - | - |
| Education (age 26) |  |  |  |  |  |  |  |  |  |  | <0.001 | 0.21 | 0.005 |
| none | 901 (34.9) | 305 (36.7) | 142 (35.9) | 163 (37.4) | 317 (36.2) | 161 (39.3) | 156 (33.5) | 279 (31.8) | 148 (31.7) | 131 (32.0) |  |  |  |
| less than O-levels | 194 (7.5) | 69 (8.3) | 26 (6.6) | 43 (9.9) | 58 (6.6) | 21 (5.1) | 37 (8.0) | 67 (7.6) | 27 (5.8) | 40 (9.8) |  |  |  |
| O-levels or equivalents | 541 (21.0) | 178 (21.4) | 64 (16.2) | 114 (26.1) | 208 (23.8) | 62 (15.1) | 146 (31.4) | 155 (17.7) | 61 (13.1) | 94 (23.0) |  |  |  |
| A-levels or equivalents | 671 (26.0) | 204 (24.5) | 106 (26.2) | 98 (22.5) | 212 (24.2) | 106 (25.9) | 106 (22.8) | 255 (29.1) | 148 (31.7) | 107 (26.2) |  |  |  |
| a degree or higher | 275 (10.7) | 75 (9.0) | 57 (26.8) | 18 (4.1) | 80 (9.1) | 60 (14.6) | 20 (4.3) | 120 (13.7) | 83 (17.8) | 37 (9.0) |  |  |  |
| Occupational class^a^ |  |  |  |  |  |  |  |  |  |  | 0.045 | 0.95 | 0.001 |
| high | 319 (11.7) | 428 (14.4) | 226 (54.6) | 202 (45.3) | 478 (9.8) | 222 (52.1) | 256 (52.4) | 478 (52.9) | 262 (54.6) | 216 (51.1) |  |  |  |
| intermediate | 975 (35.8) | 308 (35.8) | 147 (35.5) | 161 (36.1) | 347 (37.9) | 160 (37.6) | 187 (38.2) | 320 (35.4) | 170 (35.4) | 150 (35.5) |  |  |  |
| low | 1384 (50.8) | 124 (14.4) | 41 (9.9) | 83 (18.6) | 90 (52.2) | 44 (10.3) | 46 (9.4) | 105 (11.6) | 48 (10.0) | 57 (13.5) |  |  |  |
| *Health* |  |  |  |  |  |  |  |  |  |  |  |  |  |
| health conditions^b^, n (%) |  |  |  |  |  |  |  |  |  |  | 0.008 |  | 0.03 |
| 0 | 1376 (50.5) | 426 (50.7) | 228 (57.7) | 198 (44.4) | 502 (56.3) | 250 (61.0) | 252 (52.3) | 448 (50.7) | 253 (53.9) | 195 (47.1) |  |  |  |
| 1 | 898 (33.0) | 291 (34.6) | 118 (29.9) | 173 (38.8) | 296 (33.2) | 124 (30.2) | 172 (35.7) | 311 (35.1) | 156 (33.3) | 155 (37.1) |  |  |  |
| 2 | 276 (10.1) | 106 (12.6) | 46 (11.6) | 60 (13.5) | 77 (8.6) | 32 (7.8) | 45 (9.3) | 93 (10.5) | 51 (10.91) | 42 (10.1) |  |  |  |
| ≥3 | 66 (2.4) | 18 (2.1) | 3 (0.8) | 15 (3.4) | 17 (1.9) | 4 (1.0) | 12 (2.7) | 31 (3.5) | 9 (1.9) | 22 (5.3) |  |  |  |
| BMI (kg/m^2^), M (SD) | 27.34 (4.73) | 28.00 (5.21) | 27.79 (4.16) | 28.20 (6.00) | 27.14 (4.64) | 27.40 (4.03) | 26.92 (5.09) | 26.93  (4.28) | 26.86  (3.60) | 27.01  (4.94) | <0.001 | 0.006 | 0.002 |
| *Lifestyle* |  |  |  |  |  |  |  |  |  |  |  |  |  |
| Smoking status, n (%) |  |  |  |  |  |  |  |  |  |  | 0.045 | 0.87 | 0.01 |
| current | 552 (20.3) | 182 (20.4) | 87 (22.0) | 114 (25.6) | 182 (20.4) | 85 (20.7) | 97 (20.1) | 169 (19.2) | 97 (20.7) | 72 (17.4) |  |  |  |
| never or former | 2063 (75.8) | 710 (79.6) | 308 (78.0) | 332 (74.4) | 710 (79.6) | 325 (79.3) | 385 (79.9) | 713 (80.8) | 371 (79.3) | 342 (82.6) |  |  |  |
| Physical activity^c^, n (%) |  |  |  |  |  |  |  |  |  |  | 0.58 | 0.71 | 0.79 |
| none (inactive) | 1243 (47.6) | 423 (45.6) | 179 (45.3) | 224 (50.2) | 423 (45.6) | 189 (46.1) | 234 (48.5) | 417 (47.3) | 220 (47.1) | 197 (47.6) |  |  |  |
| 1-4 times/month (intermediate) | 467 (17.9) | 146 (15.7) | 79 (20.0) | 75 (16.8) | 146 (15.7) | 69 (16.8) | 77 (16.0) | 167 (19.0) | 90 (19.3) | 77 (18.6) |  |  |  |
| ≥5 times/month (active) | 904 (34.6) | 323 (34.8) | 137 (34.7) | 147 (33.3) | 323 (34.8) | 152 (37.1) | 171 (35.5) | 297 (33.7) | 157 (33.6) | 140 (33.8) |  |  |  |
| Grand mean of total milk (g/day), M (SD) | 205.55 (103.98) | - | - | - | - | - | - | - | - | - | - | - | - |
| Grand mean of full-fat milk (g/day), M (SD) | 121.59 (96.17) | 48.30 (40.09) | 51.29 (41.0) | 45.56 (39.10) | 82.54 (58.42) | 86.59 (61.0) | 78.98 (55.88) | 127.61 (103.29) | 145.50 (110.72) | 107.39 (90.14) | <0.001 | <0.001 | <0.001 |
| Grand mean of reduced-fat milk (g/day), M (SD) | 86.59  (79.41) | 51.50 (43.31) | 47.97 (42.48) | 54.74 (43.85) | 115.38 (60.80) | 109.31 (62.54) | 120.71 (58.78) | 195.44 (108.49) | 179.03 (111.67) | 214.00 (101.77) | <0.001 | <0.001 | <0.001 |
| *Other factors* |  |  |  |  |  |  |  |  |  |  |  |  |  |
| Attrition after age 53^d^, n (%) |  |  |  |  |  |  |  |  |  |  | 0.001 | 0.03 | 0.002 |
| yes | 2470 (90.4) | 773 (87.7) | 365 (86.7) | 408 (88.7) | 862 (92.9) | 400 (92.2) | 462 (93.5) | 835 (91.4) | 429 (88.5) | 406 (94.6) |  |  |  |
| no | 253 (9.6) | 108 (12.3) | 56 (13.3) | 52 (11.3) | 66 (7.1) | 34 (7.8) | 32 (6.5) | 79 (8.6) | 56 (11.5) | 23 (5.4) |  |  |  |

^a^Based on the head of household occupation (at age 53 and, if missing, at age 43 years) and categorised using the Registrar General’s Social Classification.

^b^Knee osteoarthritis and hand osteoarthritis, severe respiratory symptoms, and other disabling or life-threatening conditions.

^c^Any sports or vigorous leisure activities in the last month at age 53 years.

^d^participated at age 60-64 and/or age 69 (yes); did not participate at age 60-64 or age 69 (no)

**p*^analytic sample^***: differences by grand mean total milk thirds across key covariates in the analytic sample (n=2723)

**p*^men^***: differences by grand mean total milk thirds across key covariates in men (n=1340)

**p*^women^***: differences by grand mean total milk thirds across key covariates in women (n=1383)

**III. Supplementary multivariable analyses**

Supplementary Table 4. The risk of probable sarcopenia^a^ at age 69 years by grand mean milk intake thirds in the analytic sample: saturated models

| **Grand mean of milk in thirds** | **Saturated Model 1**:  OR (95% CI) | **p** | **Saturated Model 2:**  OR (95% CI) | **p** |
| --- | --- | --- | --- | --- |
| n | 1762 |  | 1757 |  |
| *Total milk, g/day* |  |  |  |  |
| T1 (≤153.00) | 1 |  | 1 |  |
| T2 (153.01-237.51) | 0.72 (0.44, 1.17) | 0.18 | 073 (0.45, 1.19) | 0.21 |
| T3 (≥237.52) | 1.10 (0.70, 1.73) | 0.67 | 1.09 (0.69, 1.73) | 0.7 |
| *Reduced-fat milk* |  |  |  |  |
| T1 (≤58.75) | 1 |  | 1 |  |
| T2 (58.76-145.25) | 0.60 (0.37, 0.98) | **0.04** | 0.61 (0.37, 0.996) | **0.048** |
| T3 (≥145.26) | 0.77 (0.48, 1.22) | 0.26 | 0.75 (0.47, 1.21) | 0.24 |
| *Full-fat milk* |  |  |  |  |
| T1 (≤48.22) | 1 |  | 1 |  |
| T2 (48.23-107.00) | 1.21 (0.78, 1.86) | 0.39 | 1.28 (0.83, 1.99) | 0.27 |
| T3 (≥107.01) | 0.85 (0.52, 1.41) | 0.53 | 0.85 (0.51, 1.41) | 0.25 |

^a^Based on the sex-specific cut-offs for low grip strength (<27 kg in men, and <16 kg in women).

Saturated Model 1 is Model 3 (from the main analysis) additionally adjusted for education (at age 26 years), smoking status (at age 53 years), and attrition after age 53 years.

Saturated Model 2 is further adjusted for grand mean of non-milk protein intake.

Supplementary Table 5**.** The risk of probable sarcopenia^a^ at age 69 years by grand mean milk intake thirds in the analytic sample: parsimonious and saturated model with imputed covariates^b^ and in the subsample with complete covariates^c^

| **Grand mean of milk in thirds** | **Parsimonious Model (M3)**: OR (95% CI) | **p** | **Saturated Model 1:** OR (95% CI) | **p** |
| --- | --- | --- | --- | --- |
|  | ***Imputed analysis*** |  | ***Imputed analysis*** |  |
| n | 1944 |  | 1944 |  |
| *Total milk, g/day* |  |  |  |  |
| T1 (≤153.00) | 1 |  | 1 |  |
| T2 (153.01-237.51) | 0.73 (0.46, 1.15) | 0.18 | 0.72 (0.46, 1.14) | 0.16 |
| T3 (≥237.52) | 0.99 (0.65, 1.52) | 0.98 | 1.04 (0.68, 1.59) | 0.87 |
| *Reduced-fat milk* |  |  |  |  |
| T1 (≤58.75) | 1 |  | 1 |  |
| T2 (58.76-145.25) | 0.60 (0.38, 0.94) | **0.03** | 0.60 (0.38, 0.95) | **0.03** |
| T3 (≥145.26) | 0.69 (0.44, 1.06) | 0.09 | 0.71 (0.45, 1.10) | 0.12 |
| *Full-fat milk* |  |  |  |  |
| T1 (≤48.22) | 1 |  | 1 |  |
| T2 (48.23-107.00) | 1.12 (0.74, 1.69) | 0.60 | 1.14 (0.75, 1.72) | 0.55 |
| T3 (≥107.01) | 0.87 (0.55, 1.39) | 0.57 | 0.89 (0.57, 1.42) | 0.62 |
|  | ***Complete covariates analysis*** |  | ***Complete covariates analysis*** |  |
| n | 1762 |  | 1762 |  |
| *Total milk, g/day* |  |  |  |  |
| T1 | 1 |  | 1 |  |
| T2 | 0.73 (0.54, 1.18) | 0.19 | 0.72 (0.44, 1.17) | 0.18 |
| T3 | 1.06 (0.68, 1.66) | 0.79 | 1.10 (0.70, 1.73) | 0.67 |
| *Reduced-fat milk* |  |  |  |  |
| T1 | 1 |  | 1 |  |
| T2 | 0.61 (0.37, 0.98) | **0.04** | 0.60 (0.37, 0.98) | **0.04** |
| T3 | 0.75 (0.47, 1.19) | 0.22 | 0.77 (0.48, 1.22) | 0.26 |
| *Full-fat milk* |  |  |  |  |
| T1 | 1 |  | 1 |  |
| T2 | 1.19 (0.77, 1.82) | 0.43 | 1.21 (0.78, 1.86) | 0.39 |
| T3 | 0.84 (0.51, 1.38) | 0.48 | 0.85 (0.51, 1.41) | 0.53 |

^a^Based on the sex-specific cut-offs for low grip strength (<27 kg in men, and <16 kg in women).

^b^Imputed covariates: occupational class (n = 45, reference value (low)), BMI (n = 125, sex-specific mean), number of health conditions (n =108, median), leisure-time physical activity (n = 109, reference value (low), highest educational level attained (n = 141, reference value (low)), and smoking status (n = 108, reference value (never/former).

^c^2445 participants in the analytic sample (89.8%) had complete data for covariates (169 (6.2%) participants had 1 covariate with missing data, 1 (0.04%) participant had 2 covariates missing, 1 (0.04%) had 3 covariates missing, 76 (2.8%) had 4 covariates missing, 29 (1.1%) had 5, and 2 (0.1%) participants had all 6 covariates with missing data).

Parsimonious model (Model 3 from the main analysis) adjusted for occupational class (at age 53 or age 43 if missing at age 53 years), health and lifestyle variables (BMI, health conditions, and leisure-time physical activity).

Saturated Model 1 is Model 3 additionally adjusted for education (at age 26 years), smoking status (at age 53 years), and attrition after age 53 years.

Supplementary Table 6. β estimates of mixed models for muscle strength (grip strength) decline^a^ from age 53 to 69 years by grand mean milk intake^b^ from mid- to late life in men and women in the analytic sample: saturated models

| **Exposure** | **Effect** | **Men** | | |  | **Women** | | |  |
| --- | --- | --- | --- | --- | --- | --- | --- | --- | --- |
|  |  | **Saturated Model 1** | **p** | **Saturated Model 2** | **p** | **Saturated Model 1** | **p** | **Saturated Model 2** | **p** |
|  |  | β (95% CI) |  |  |  | β (95% CI) |  |  |  |
| *Total milk* | Time | -0.44 (-0.53, -0.35) | <0.001 | -0.44 (-0.53, -0.36) | <0.001 | -0.26 (-0.32, -0.20) | <0.001 | -0.26 (-0.32, -0.20) | <0.001 |
| *g/day* | T1 (≤153.0) (ref) | 0 |  | 0 |  | 0 |  | 0 |  |
|  | T2 (153.01-237.51) | 1.13 (-0.60, 2.86) | 0.20 | 1.06 (-0.68, 2.79) | 0.23 | 0.17 (-0.88, 1.23) | 0.75 | 0.11 (-0.95, 1.18) | 0.84 |
|  | T3 (≥237.52) | 1.66 (-0.01, 3.33) | 0.052 | 1.49 (-0.20, 3.17) | 0.08 | -0.30 (-1.40, 0.80) | 0.59 | -0.34 (-1.45, 0.77) | 0.55 |
|  | Slope |  |  |  |  |  |  |  |  |
|  | T1 × Time (ref) | 0 |  | 0 |  | 0 |  | 0 |  |
|  | T2 × Time | -0.05 (-0.16, 0.07) | 0.44 | -0.05 (-0.16, 0.07) | 0.44 | -0.03 (-0.10, 0.05) | 0.51 | -0.03 (-0.10, 0.05) | 0.50 |
|  | T3 × Time | -0.07 (-0.18, 0.05) | 0.24 | -0.06 (-0.18, 0.05) | 0.28 | 0.02 (-0.06, 0.09) | 0.67 | 0.02 (-0.06, 0.10) | 0.64 |
| *Reduced-fat milk* | Time | -0.45 (-0.66, 2.81) | <0.001 | -0.45 (-0.54, -0.36) | <0.001 | -0.32 (-0.40, -0.26) | <0.001 | -0.33 (-0.40, -0.26) | <0.001 |
| *g/day* | T1 (≤58.75) (ref) | 0 |  | 0 |  | 0 |  | 0 |  |
|  | T2 (58.76-145.25) | 1.04 (-0.67, 2.76) | 0.23 | 0.93 (-0.81, 2.66) | 0.3 | -0.45 (-1.61, 0.70) | 0.44 | -0.57 (-1.74, 0.60) | 0.34 |
|  | T3 (≥145.26) | 1.08 (-0.66, 2.80) | 0.22 | 0.92 (-0.84, 2.68) | 0.31 | -0.67 (-1.82, 0.49) | 0.26 | -0.78 (-1.95, 0.38) | 0.19 |
|  | Slope |  |  |  |  |  |  |  |  |
|  | T1 × Time (ref) | 0 |  | 0 |  | 0 |  | 0 |  |
|  | T2 × Time | -0.04 (-0.16, 0.08) | 0.54 | -0.05 (-0.17, 0.07) | 0.43 | 0.08 (-0.01, 0.16) | 0.07 | 0.08 (-0.01, 0.16) | 0.07 |
|  | T3 × Time | -0.04 (-0.16, 0.07) | 0.48 | -0.04 (-0.16, 0.08) | 0.49 | 0.09 (0.005, 0.17) | **0.04** | 0.09 (0.01, 0.17) | **0.03** |
| *Full-fat milk* | Time | -0.51 (-0.59, -0.42) | <0.001 | -0.51 (-0.59, -0.42) | <0.001 | -0.25 (-0.30, -0.20) | <0.001 | -0.25 (-0.30, -0.20) | <0.001 |
| *g/day* | T1 (≤48.22) (ref) | 0 |  | 0 |  | 0 |  | 0 |  |
|  | T2 (48.23-107.0) | -0.90 (-2.61, 0.81) | 0.3 | -1.09 (-2.81, 0.64) | 0.22 | 0.38 (-0.63, 1.38) | 0.46 | 0.33 (-0.68, 1.34) | 0.52 |
|  | T3 (≥107.01) | 1.54 (-0.17, 3.25) | 0.08 | 1.47 (-0.24, 3.19) | 0.09 | -0.04 (-1.17, 1.09) | 0.95 | 0.02 (-1.11, 1.16) | 0.97 |
|  | Slope |  |  |  |  |  |  |  |  |
|  | T1 × Time (ref) | 0 |  | 0 |  | 0 |  | 0 |  |
|  | T2 × Time | 0.08 (-0.03, 0.20) | 0.16 | 0.08 (-0.03, 0.19) | 0.17 | -0.03 (-0.10, 0.04) | 0.4 | -0.03 (-0.10, 0.04) | 0.37 |
|  | T3 × Time | -0.01 (-0.13, 0.10) | 0.83 | -0.01 (-0.13, 0.11) | 0.86 | 0.001 (-0.08, 0.08) | 0.99 | -0.001 (-0.08, 0.08) | 0.97 |

ref, reference.

^a^Maximum grip strength measured four times in both hands for each assessment.

^b^Grand mean milk thirds of total, reduced-fat, and full-fat milk from age 36 (1982) to age 60-64 (2006/2011) in participants who had complete data for milk intake (g/day) for at least 3 days of a 5-day diet diary at each diet assessment.

Negative coefficients for grip strength indicate poorer performance.

Saturated Models 1 are Model 3 (from the main analysis) additionally adjusted for education (at age 26 years), smoking status (at age 53 years), and attrition after age 53 years.

Saturated Models 2 are further adjusted for grand mean of non-milk protein intake.

Supplementary Table 7. β estimates of mixed models for muscle strength (grip strength) decline^a^ from age 53 to 69 years by mean milk intake^b^ across adulthood (age 36 to 60-64 years) in men and women in the analytic sample: imputed analyses and in the subsample with complete covariates^c^

| **Exposure** | **Effect** | **Model 3 (parsimonious model)** | **p** | **Saturated Model 1** | **p** |
| --- | --- | --- | --- | --- | --- |
|  |  | ***Imputed analysis*** |  | ***Imputed analysis*** |  |
| Men |  | β (95% CI) |  | β (95% CI) |  |
| *Total milk* | Time | -0.45 (-0.53, -0.36) | <0.001 | -0.46 (-0.54, -0.37) | <0.001 |
| *g/day* | T1 (≤152.0) (ref) | 0 |  | 0 |  |
|  | T2 (153.01-237.51) | 1.30 (-0.36, 2.97) | 0.12 | 1.22 (-0.44, 2.89) | 0.15 |
|  | T3 (≥237.52) | 1.85 (0.23, 3.47) | **0.03** | 1.83 (0.21, 3.45) | **0.03** |
|  | Slope |  |  |  |  |
|  | T1 × Time (ref) | 0 |  | 0 |  |
|  | T2 × Time | -0.02 (-0.14, 0.09) | 0.67 | -0.02 (-0.13, 0.09) | 0.73 |
|  | T3 × Time | -0.05 (-0.16, 0.06) | 0.34 | -0.05 (-0.16, 0.06) | 0.36 |
| *Reduced-fat milk* | Time | -0.44 (-0.52, -0.35) | <0.001 | -0.45 (-0.54, -0.37) | <0.001 |
| *g/day* | T1 (≤58.75) (ref) | 0 |  | 0 |  |
|  | T2 (58.76-145.25) | 1.42 (-0.21, 3.05) | 0.09 | 1.17 (-0.39, 3.92) | 0.13 |
|  | T3 (≥145.26) | 1.30 (-0.35, 2.95) | 0.12 | 1.19 (-0.49, 2.87) | 0.17 |
|  | Slope |  |  |  |  |
|  | T1 × Time (ref) | 0 |  | 0 |  |
|  | T2 × Time | -0.06 (-0.17, 0.06) | 0.32 | -0.05 (-0.16, 0.07) | 0.42 |
|  | T3 × Time | -0.04 (-0.15, 0.07) | 0.47 | -0.03 (-0.15, 0.82) | 0.59 |
| *Full-fat milk* | Time | -0.51 (-0.59, -0.43) | <0.001 | -0.52 (-0.60, -0.44) | <0.001 |
| *g/day* | T1 (≤48.22) (ref) | 0 |  | 0 |  |
|  | T2 (48.23-107.0) | -0.85 (-2.49, 0.79) | 0.31 | -0.90 (-2.54, 0.74) | 0.28 |
|  | T3 (≥107.01) | 1.32 (-0.33, 2.96) | 0.12 | 1.40 (-0.24, 3.05) | 0.09 |
|  | Slope |  |  |  |  |
|  | T1 × Time (ref) | 0 |  | 0 |  |
|  | T2 × Time | 0.09 (-0.02, 0.20) | 0.10 | 0.10 (-0.01, 0.20) | 0.08 |
|  | T3 × Time | 0.02 (-0.09, 0.13) | 0.78 | 0.01 (-0.10, 0.12) | 0.85 |
| Women |  |  |  |  |  |
| *Total milk* | Time | -0.25 (-0.30, -0.20) | <0.001 | -0.26 (-0.31, -0.21) | <0.001 |
| *g/day* | T1 (ref) | 0 |  | 0 |  |
|  | T2 | 0.18 (-0.83, 1.19) | 0.72 | 0.14 (-0.87, 1.15) | 0.79 |
|  | T3 | -0.36 (-1.40, 0.68) | 0.50 | -0.50 (-1.55, 0.54) | 0.34 |
|  | Slope |  |  |  |  |
|  | T1 × Time (ref) | 0 |  | 0 |  |
|  | T2 × Time | -0.03 (-0.1, 0.05) | 0.49 | -0.02 (-0.09, 0.05) | 0.55 |
|  | T3 × Time | 0.02 (-0.06, 0.09) | 0.65 | 0.02 (-0.05, 0.10) | 0.58 |
| *Reduced-fat milk* | Time | -0.31 (-0.38, -0.25) | <0.001 | -0.33 (-0.39, -0.26) | <0.001 |
| *g/day* | T1 (ref) | 0 |  | 0 |  |
|  | T2 | -0.39 (-1.47, 0.70) | 0.49 | -0.55 (-1.65, 0.55) | 0.33 |
|  | T3 | -0.66 (-1.74, 0.41) | 0.23 | -0.64 (-1.87, 0.58) | 0.33 |
|  | Slope |  |  |  |  |
|  | T1 × Time (ref) | 0 |  | 0 |  |
|  | T2 × Time | 0.07 (-0.01, -0.15) | 0.09 | 0.08 (-0.002, 0.16) | 0.06 |
|  | T3 × Time | 0.08 (0.001, 0.16) | **0.04** | 0.09 (0.001, 0.17) | **0.02** |
| *Full-fat milk* | Time | -0.25 (-0.29, -0.20) | <0.001 | -0.25 (-0.30, -0.20) | <0.001 |
| *g/day* | T1 (ref) | 0 |  | 0 |  |
|  | T2 | 0.23 (-0.73, 1.18) | 0.64 | 0.20 (-0.75, 1.16) | 0.67 |
|  | T3 | -0.27 (-1.34, 0.80) | 0.62 | -0.20 (-1.27, 0.88) | 0.72 |
|  | Slope |  |  |  |  |
|  | T1 × Time | 0 |  | 0 |  |
|  | T2 × Time | -0.03 (-0.09, 0.04) | 0.44 | -0.03 (-0.10, 0.04) | 0.42 |
|  | T3 × Time | 0.002 (-0.75, 0.08) | 0.96 | -0.04 (-0.08, 0.07) | 0.92 |
|  |  | ***Complete covariate analysis*** |  | ***Complete covariate analysis*** |  |
| Men |  |  |  |  |  |
| *Total milk* | Time | -0.44 (-0.52, -0.35) | <0.001 | -0.44 (-0.53, -0.35) | <0.001 |
| *g/day* | T1 (ref) | 0 |  | 0 |  |
|  | T2 | 1.15 (-0.58, 2.88) | 0.19 | 1.13 (-0.60, 2.86) | 0.20 |
|  | T3 | 1.67 (-0.01, 3.34) | 0.051 | 1.66 (-0.01, 3.34) | 0.052 |
|  | Slope |  |  |  |  |
|  | T1 × Time (ref) | 0 |  |  |  |
|  | T2 × Time | -0.05 (-0.16, 0.07) | 0.66 | -0.05 (-0.16, 0.07) |  |
|  | T3 × Time | -0.07 (-0.18, 0.05) | 0.33 | -0.07 (-0.18, 0.05) |  |
| *Reduced-fat milk* | Time | -0.45 (-0.53, -0.36) | <0.001 | -0.45 (-0.54, -0.36) | <0.001 |
| *g/day* | T1 (ref) | 0 |  | 0 |  |
|  | T2 | 1.09 (-0.60, 2.78) | 0.20 | 1.05 (-0.67, 2.76) | 0.23 |
|  | T3 | 1.06 (-0.64, 2.77) | 0.22 | 1.08 (-0.66, 2.81) | 0.22 |
|  | Slope |  |  |  |  |
|  | T1 × Time | 0 |  | 0 |  |
|  | T2 × Time | -0.05 (-0.16, 0.07) | 0.42 | -0.04 (-0.16, 0.08) |  |
|  | T3 × Time | -0.04 (-0.16, 0.08) | 0.50 | -0.04 (-0.16, 0.08) | 0.54 |
| *Full-fat milk* | Time | -0.50 (-0.58, 0.41) | <0.001 | -0.51 (-0.59, 0.42) | <0.001 |
| *g/day* | T1 (ref) | 0 |  | 0 |  |
|  | T2 | -0.87 (-2.58, 0.83) | 0.31 | -0.91 (-2.61, 0.81) | 0.30 |
|  | T3 | 1.51 (-0.20, 3.22) | 0.08 | 1.54 (-0.17, 3.25) | 0.08 |
|  | Slope |  |  |  |  |
|  | T1 × Time | 0 |  | 0 |  |
|  | T2 × Time | -0.87 (-2.58, 0.83) | 0.31 | 0.08 (-0.03, 0.20) | 0.16 |
|  | T3 × Time | -0.01 (-0.13, 0.11) | 0.88 | -0.01 (-0.13, 0.10) | 0.83 |
| Women |  |  |  |  |  |
| *Total milk* | Time | -0.25 (-0.31, -0.19) | <0.001 | -0.26 (-0.32, -0.20) | <0.001 |
| *g/day* | T1 (ref) | 0 |  | 0 |  |
|  | T2 | 0.23 (-0.83, 1.29) | 0.67 | 0.17 (-0.89, 1.23) | 0.75 |
|  | T3 | -0.13 (-1.23, 0.96) | 0.81 | -0.30 (-1.40, 0.80) | 0.59 |
|  | Slope |  |  |  |  |
|  | T1 × Time | 0 |  | 0 |  |
|  | T2 × Time | -0.03 (-0.10, 0.05) | 0.46 | -0.03 (-0.10, 0.05) | 0.51 |
|  | T3 × Time | 0.01 (-0.06, 0.09) | 0.74 | 0.02 (-0.06, 0.09) | 0.67 |
| *Reduced-fat milk* | Time | -0.31 (-0.38, -0.25) | <0.001 | -0.33 (-0.40, -0.26) | <0.001 |
|  | T1 (ref) | 0 |  | 0 |  |
|  | T2 | -0.28 (-1.43, 0.87) | 0.63 | -0.45 (-1.61, 0.70) | 0.44 |
|  | T3 | -0.43 (-1.56, 0.71) | 0.46 | -0.67 (-1.82, 0.49) | 0.26 |
|  | Slope |  |  |  |  |
|  | T1 × Time | 0 |  | 0 |  |
|  | T2 × Time | 0.07 (-0.01, 0.15) | 0.10 | 0.08 (-0.01, 0.16) | 0.07 |
|  | T3 × Time | 0.08 (-0.004, 0.16) | 0.06 | 0.09 (0.005, 0.17) | **0.04** |
| *Full-fat milk* | Time | -0.25 (-0.30, -0.20) | <0.001 | -0.25 (-0.30, -0.20) | <0.001 |
| *g/day* | T1 (ref) | 0 |  | 0 |  |
|  | T2 | 0.41 (-0.60, 1.41) | 0.43 | 0.38 (-0.63, 1.38) | 0.46 |
|  | T3 | -0.12 (-1.24, 1.01) | 0.84 | -0.04 (-1.17, 1.09) | 0.95 |
|  | Slope |  |  |  |  |
|  | T1 × Time | 0 |  | 0 |  |
|  | T2 × Time | -0.03 (-0.10, 0.04) | 0.41 | -0.03 (-0.10, 0.04) | 0.40 |
|  | T3 × Time | 0.01 (-0.07, 0.09) | 0.87 | 0.001 (-0.08, 0.08) | 0.99 |

ref, reference.

^a^Maximum grip strength measured four times in both hands for each assessment.

^b^Mean milk intake in thirds of total, reduced-fat, and full-fat milk from age 36 (1982) to age 60-64 (2006/2011) in participants who had complete data for milk intake (g/day) for at least 3 days of a 5-day diet diary at each diet assessment.

^c^2445 participants in the analytic sample (89.8%) had complete data for covariates (169 (6.2%) participants had 1 covariate with missing data, 1 (0.04%) participant had 2 covariates missing, 1 (0.04%) had 3 covariates missing, 76 (2.8%) had 4 covariates missing, 29 (1.1%) had 5, and 2 (0.1%) participants had all 6 covariates with missing data).

Model 3 (parsimonious model) included liner trend of time (age centred at 53 years), random intercepts and slopes, and was adjusted for grand mean milk intake thirds (total, reduced-fat, and full-fat milk, respectively) and interaction terms (mean milk intake thirds × time), socio-demographic (social class), health and lifestyle variables (health conditions, BMI, and leisure-time physical activity).

Saturated Model 1 is Model 3 further adjusted for highest level of education attained, smoking status, and attrition after age 53 years.

Negative coefficients for grip strength indicate poorer performance.

Imputed covariates: occupational class (n = 45, reference value (low)), BMI (n = 125, sex-specific mean), number of health conditions (n =108, median), leisure-time physical activity (n = 109, reference value (low), highest educational level attained (n = 141, reference value (low)), and smoking status (n = 108, reference value (never/former)).

Supplementary Table 8. Linear regression summary of effect sizes^a^ for milk intake^b^ predicting contemporaneous grip strength^c^ (kg) in the analytic sample

|  | **Milk type** | **Model** | **Effect size (95% CI)** | **p** |
| --- | --- | --- | --- | --- |
| Grip strength at age 53 (kg) | *Total milk* | Model 1 | -0.02 (-0.15, 0.11) | 0.76 |
|  |  | Model 2 | 0.01 (-0.08, 0.10) | 0.83 |
|  |  | Model 3 | 0.01 (-0.09, 0.10) | 0.88 |
|  | *Reduced-fat milk* | Model 1 | -0.02 (-0.12, 0.09) | 0.74 |
|  |  | Model 2 | 0.02 (-0.06, 0.10) | 0.57 |
|  |  | Model 3 | 0.01 (-0.07, 0.09) | 0.77 |
|  | *Full- fat milk* | Model 1 | -0.04 (-0.19, 0.11) | 0.59 |
|  |  | Model 2 | -0.06 (-0.17, 0.05) | 0.28 |
|  |  | Model 3 | -0.05 (-0.17, 0.06) | 0.35 |
| Grip strength at age 60-64 (kg) | *Total milk* | Model 1 | 0.17 (0.05, 0.29) | **0.005** |
|  |  | Model 2 | 0.10 (0.02, 0.19) | **0.02** |
|  |  | Model 3 | 0.09 (0.002, 0.18) | **0.04** |
|  | *Reduced-fat milk* | Model 1 | 0.09 (-0.02, 0.20) | 0.11 |
|  |  | Model 2 | 0.09 (0.01, 0.17) | **0.03** |
|  |  | Model 3 | 0.08 (-0.002, 0.16) | 0.055 |
|  | *Full- fat milk* | Model 1 | 0.11 (-0.05, 0.26) | 0.173 |
|  |  | Model 2 | -0.004 (-0.12-, 0.11) | 0.95 |
|  |  | Model 3 | -0.012 (-0.13, 0.10) | 0.055 |

^a^Standardised beta coefficients (β).

^b^Milk intake of total, reduced-fat, and full-fat milk from age 53 (1999) and age 60-64 (2006/2011) in participants who had complete data for milk intake (g/day) for at least 3 days of a 5-day diet diary at each diet assessment. Positive skew of each milk intake was corrected as √x (x representing original value).

^c^Maximum grip strength measured four times in both hands for each assessment.

Model 1 was unadjusted.

Model 2 was adjusted for milk intake (total, reduced-fat, and full-fat milk; continuous), socio-demographic (sex and social class), health and lifestyle variables (health conditions, BMI, and leisure-time physical activity).

Model 3 was additionally adjusted for education (at age 26 years), smoking status, and attrition after age 53 years.

Supplementary Table 9. Linear regression summary of effect sizes^a^ for milk intake^b^ at ages 36, 43, 53 and 60-64 years predicting grip strength^c^ (kg) at age 69 years in the analytic sample

| **Milk type** | **Age** | **Model** | **Effect size (95% CI)** | **p** |
| --- | --- | --- | --- | --- |
| *Total milk* | 36 | Model 1 | 0.30 (0.17, 0.42) | **<0.001** |
|  |  | Model 2 | 0.09 (0.005, 0.18) | **0.04** |
|  |  | Model 3 | 0.08 (-0.01, 0.17) | 0.08 |
| *Reduced-fat milk* | 36 | Model 1 | -0.32 (-0.51, -0.14) | **0.001** |
|  |  | Model 2 | -0.002 (-0.13, 0.12) | 0.97 |
|  |  | Model 3 | 0.01 (-0.11, 0.14) | 0.83 |
| *Full-fat milk* | 36 | Model 1 | 0.34 (0.22, 0.46) | **<0.001** |
|  |  | Model 2 | 0.07 (-0.01, 0.15) | 0.1 |
|  |  | Model 3 | 0.05 (-0.03, 0.14) | 0.21 |
| *Total milk* | 43 | Model 1 | 0.16 (0.04, 0.28) | **0.007** |
|  |  | Model 2 | 0.09 (0.01, 0.17) | **0.02** |
|  |  | Model 3 | 0.08 (0.000, 0.16) | **0.049** |
| *Reduced-fat milk* | 43 | Model 1 | -0.11 (-0.18, -0.03) | **0.004** |
|  |  | Model 2 | -0.01 (-0.06, 0.04) | 0.74 |
|  |  | Model 3 | -0.01 (-0.06, 0.04) | 0.77 |
| *Full-fat milk* | 43 | Model 1 | 0.24 (0.16, 0.32) | **<0.001** |
|  |  | Model 2 | 0.07 (0.01, 0.12) | **0.02** |
|  |  | Model 3 | 0.06 (0.006, 0.12) | **0.03** |
| *Total milk* | 53 | Model 1 | -0.02 (-0.13, 0.09) | 0.73 |
|  |  | Model 2 | -0.001 (-0.08, 0.07) | 0.97 |
|  |  | Model 3 | -0.004 (-0.08, 0.07) | 0.91 |
| *Reduced-fat milk* | 53 | Model 1 | 0.001 (-0.09, 0.10) | 0.99 |
|  |  | Model 2 | -0.02 (-0.08, 0.05) | 0.6 |
|  |  | Model 3 | -0.01 (-0.08, 0.05) | 0.7 |
| *Full-fat milk* | 53 | Model 1 | -0.09 (-0.23, 0.05) | 0.19 |
|  |  | Model 2 | 0.02 (-0.07, 0.12) | 0.61 |
|  |  | Model 3 | 0.004 (-0.09, 0.1) | 0.94 |
| *Total milk* | 60-64 | Model 1 | 0.13 (0.03, 0.23) | **0.01** |
|  |  | Model 2 | 0.07 (0.01, 0.14) | **0.03** |
|  |  | Model 3 | 0.07 (-0.002, 0.13) | 0.06 |
| *Reduced-fat milk* | 60-64 | Model 1 | 0.09 (-0.002, 0.18) | 0.06 |
|  |  | Model 2 | 0.08 (0.02, 0.14) | **0.009** |
|  |  | Model 3 | 0.08 (0.02, 0.14) | **0.01** |
| *Full-fat milk* | 60-64 | Model 1 | 0.05 (-0.08, 0.17) | 0.48 |
|  |  | Model 2 | -0.03 (-0.12, 0.06) | 0.51 |
|  |  | Model 3 | -0.05 (-0.13, 0.04) | 0.31 |

^a^Standardised beta coefficients (β).

^b^Milk intake of total, reduced-fat, and full-fat milk from age 36 (1982), 43 (1989), 53 (1999) and 60-64 (2006/2011) years in participants who had complete data for milk intake (g/day) for at least 3 days of a 5-day diet diary at each diet assessment. Positive skew of each milk intake was corrected as √x (x representing the original continuous variable).

^c^Maximum grip strength measured four times in both hands.

Model 1 was unadjusted.

Model 2 was adjusted for milk intake (total, reduced-fat, and full-fat milk; continuous), socio-demographic (sex and social class), health and lifestyle variables (health conditions, BMI, and leisure-time physical activity).

Model 3 was additionally adjusted for education (at age 26 years), smoking status, and attrition after age 53 years.

Supplementary Table 10. Linear regression summary of effect sizes^a^ for grand mean milk intake^b^ predicting grip strength (kg) at age 69 years in the analytic sample

| **Milk type** | **Model** | **Effect size (95% CI)** | **p** |
| --- | --- | --- | --- |
| *Total milk* | Model 1 | 0.16 (0.04, 0.28) | **0.01** |
|  | Model 2 | 0.08 (-0.002, 0.16) | 0.055 |
|  | Model 3 | 0.06 (-0.02, 0.15) | 0.16 |
| *Reduced-fat milk* | Model 1 | -0.06 (-0.16, 0.03) | 0.2 |
|  | Model 2 | 0.03 (-0.039, 0.1) | 0.41 |
|  | Model 3 | 0.03 (-0.04, 0.1) | 0.36 |
| *Full-fat milk* | Model 1 | 0.25 (0.14, 0.36) | **<0.001** |
|  | Model 2 | 0.07 (-0.004, 0.15) | 0.06 |
|  | Model 3 | 0.06 (-0.02, 0.13) | 0.16 |

^a^Standardised beta coefficients (β).

^b^Grand mean of total, reduced-fat, and full-fat milk intake from age 36 (1982) to age 60-64 (2006/2011) years in participants who had complete data for milk intake (g/day) for at least 3 days of a 5-day diet diary at each diet assessment. Positive skew of each milk intake was corrected as √x (x representing original value).

^c^Maximum grip strength measured four times in both hands.

Model 1 was unadjusted.

Model 2 was adjusted for milk intake (total, reduced-fat, and full-fat milk; continuous), socio-demographic (sex and social class), health and lifestyle variables (health conditions, BMI, and leisure-time physical activity).

Model 3 was additionally adjusted for education (at age 26 years), smoking status, and attrition after age 53 years.

**IV. Supplementary figures**


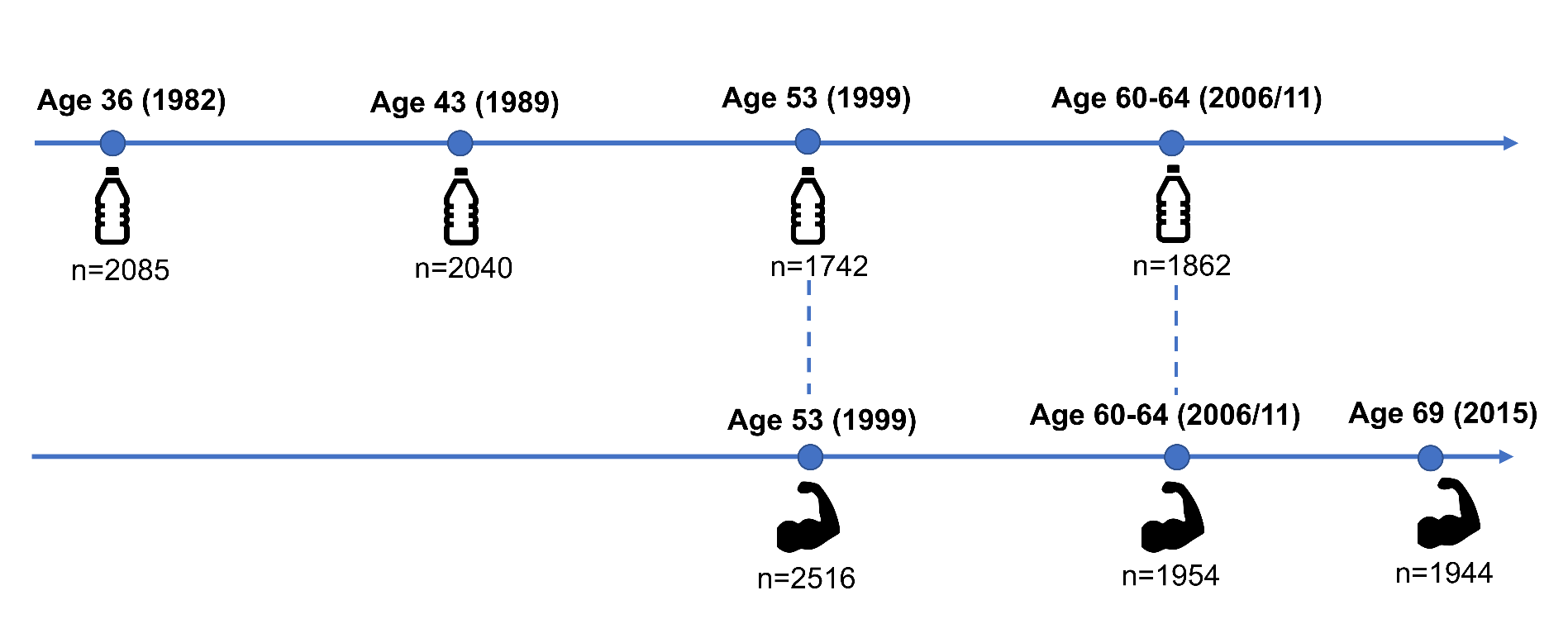


**Supplementary Figure 1** Timeline of exposure (milk) and outcome (grip strength) in the MRC National Survey of Health and Development


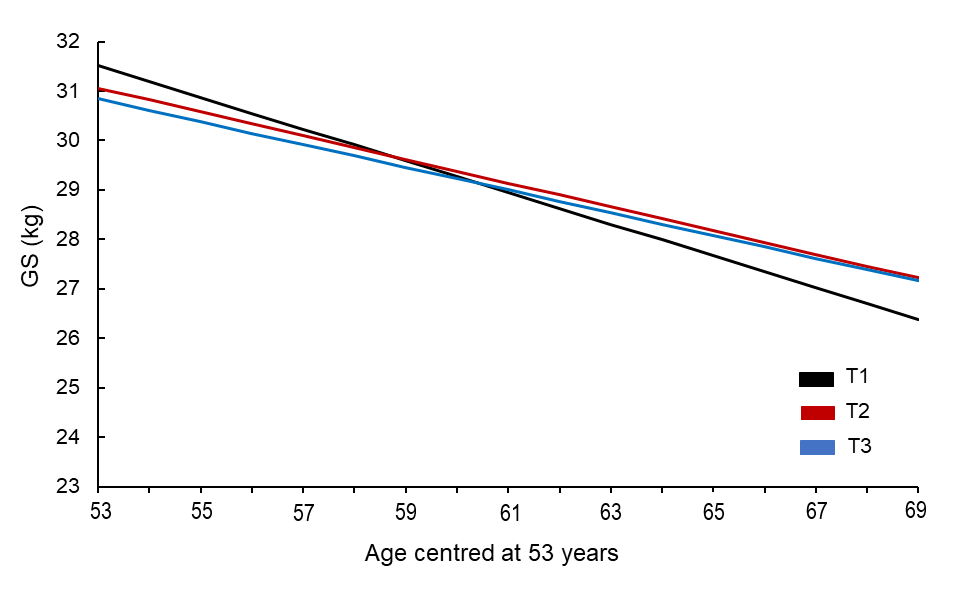


**Supplementary Figure 2** Estimated 16-year trajectory of grip strength by grand mean reduced-fat milk thirds across adulthood in women in the analytic sample.

In the Saturated Model 1 additionally adjusted for education, smoking status, and attrition at age 53 years (Supplementary Table 6), the highest third (T3) of grand mean reduced-fat milk (≥145.26g/day; blue line) was associated with less steep grip strength decline (GS; kg) over 16 years compared with T1 (≤58.75g/day; black line) in women.

**V. Supplementary References**

S1. Dodds RM, Roberts HC, Cooper C, Sayer AA. The epidemiology of sarcopenia. *J Clin Densitom* 2015;***18***:461–466.

S2. Shaw SC, Dennison EM, Cooper C. Epidemiology of Sarcopenia: Determinants Throughout the Lifecourse. *Calcif Tissue Int* 2017;**101**:229–247.

S3. Dodds RM, Granic A, Robinson SM, Sayer AA. Sarcopenia, long-term conditions, and multimorbidity: findings from UK Biobank participants. *J Cachexia Sarcopenia Muscle* 2020;**11**:62–68.

S4. Hurst C, Murray JC, Granic A, Hillman SJ, Cooper R, Sayer AA, Robinson SM, Dodds RM. Long-term conditions, multimorbidity, lifestyle factors and change in grip strength over 9 years of follow-up: Findings from 44,315 UK biobank participants. *Age Ageing* 2021;**50**:2222–2229.

S5. Robinson SM, Westbury LD, Ward K, Syddall H, Cooper R, Cooper C, Sayer AA. Is lifestyle change around retirement associated with better physical performance in older age?: insights from a longitudinal cohort. *Eur J Ageing* 2021;**18**:513–521.
